# Hospital-to-hospice transfers in Germany: analyzing the impact of age and gender at the care transition interface

**DOI:** 10.3389/fonc.2026.1743124

**Published:** 2026-03-26

**Authors:** Christiane von Sass, Birgitt van Oorschot, Tabea Sammer, Martina Kern, Lukas Radbruch, Marc Rütters, Nazife Dinc, Marcel A. Kamp

**Affiliations:** 1 Brandenburg Medical School Theodor Fontane and Faculty of Health Sciences Brandenburg, Rüdersdorf, Germany; 2 Independent Researcher, Rangsdorf, Germany; 3 Department of Palliative Medicine, Franziskus Hospital Berlin, Berlin, Germany; 4 Ansprechstellen im Land NRW zur Palliativversorgung, Hospizarbeit und Angehörigenbegleitung (ALPHA) Rheinland, Bonn, Germany; 5 Klinik und Poliklinik für Palliativmedizin, Universitätsklinikum Bonn, Bonn, Germany; 6 Department of Neurosurgery, Jena University Hospital, Friedrich-Schiller-University, Jena, Germany; 7 Department of Palliative and Neuropalliative Care, Immanuel Clinic Rüdersdorf, University Hospital of the Brandenburg Medical School Theodor Fontane, Rüdersdorf, Germany

**Keywords:** end of life disparities, hospital to hospice interface, inpatient hospice care, palliative care, regional bed density, supply driven care

## Abstract

**Background:**

Hospice care models vary worldwide due to differences in healthcare systems, cultural attitudes, and resource availability. In Germany, inpatient hospice care plays a vital role in the treatment of critically ill patients, providing inpatient end-of-life care if home care is not possible. The transition between different care settings is a critical interface. This study analyzes aggregated 2023 hospital data to assess the frequency and characteristics of direct transfers from hospitals to inpatient hospices and evaluates the impact of regional infrastructure on these transitions.

**Methods:**

We conducted a cross-sectional analysis of aggregated data from all German hospital stays in 2023, using nationwide hospital billing data reported under §21 of the Hospital Remuneration Act. The study included adult cases and employed linear regression to examine the association between regional bed density and transfer rates.

**Results:**

In 2023, 15,656 cases resulted in a transfer to an inpatient hospice. This transfer rate varied by region, with the highest rates in Saarland and Brandenburg (0.2% and 0.19%, respectively) and the lowest in Bavaria (0.05%). Hospital to hospice transfers accounted for 32.7% of all hospice admissions. Over 75% of hospice transfers were aged 65 or older. Female patients comprised 53.6% of the hospice transfer cohort, reflecting a significant disparity likely linked to limited informal spousal support. Oncological diseases were the primary diagnosis in 68.8% of cases. Transfer rates correlated strongly with regional hospice bed density (R^2^ = 0.65) but showed no association with outpatient palliative care density or socioeconomic indicators.

**Conclusions:**

Hospital-to-hospice transfers in Germany follow a supply-driven logic where regional capacity outweighs clinical demand. These transitions primarily involve elderly patients and women vulnerable to the collapse of informal support networks. As healthcare policy seeks to integrate inpatient and outpatient sectors, these findings serve as a benchmark for transitioning toward a needs-oriented planning model to ensure equitable end-of-life access.

## Introduction

Hospice care varies worldwide due to differences in healthcare systems, cultural attitudes, and resource availability. In the United States, hospice care often relies on Medicare funding and includes multi-professional care (1, 2), while Canada provides public funding with varying coverage (3–6). The United Kingdom maintains a long-established, charity-based hospice movement funded by the National Health Service as well as by charitable donations (5, 7). However, hospice care in many developing countries struggles with limited resources and infrastructural challenges (5).

Germany’s palliative care system integrates general and specialized services, ensuring timely and continuous care for patients with complex symptoms (8, 9). In contrast to specialized outpatient palliative care, hospice care excludes further life-prolonging therapies, such as anti-tumor therapy. Inpatient hospices support individuals with advanced illnesses who can no longer be cared for at home. According to the updated framework agreement, admission to an inpatient hospice requires a medical certificate confirming a terminal illness that is progressing toward death within weeks or months, for which curative treatment is no longer possible. Additionally, it must be established that necessary palliative-medical and nursing care cannot be sufficiently or feasibly provided within the patient’s domestic or familiar environment (10, 11). Specialized palliative nurses deliver comprehensive care, while medical assistance is provided by general practitioners or specialized outpatient palliative care (SAPV) teams. The 2015 Hospice and Palliative Care Act (HPG) strengthened financial support and service coordination, increasing reimbursement rates and expanding outpatient funding (9). While insurance covers most inpatient hospice costs, charitable donations contribute to the remaining expenses. Outpatient hospice care, primarily supported by trained volunteers, provides psychosocial assistance, easing the burden on professional caregivers.

The transition between standard medical care and palliative or hospice care is a critical phase in the healthcare continuum. A qualitative study conducted in the United States examined the interface between hospital treatment and hospice care, identifying potential challenges in this transition (12). A 2016 mixed-methods retrospective analysis conducted in Augsburg and Bonn specifically identified pain-related complications occurring at healthcare interface points (13). While several high-quality studies, including those from German-speaking countries, have analyzed transitions between health care settings in the last year of life, no quantitative research has yet investigated hospital-to-hospice transfers (14, 15). This study aims to analyze the number of hospital cases transferred to hospice care and to further characterize this cohort.

## Methods

### Ethical approval and data accessibility

The study complied with the ethical standards set in the 1964 Declaration of Helsinki and its subsequent revisions. Approval for the study protocol was granted by the institutional and local ethics committee at Friedrich Schiller University Jena (Study ID: 2025-3696-BO-D) and Brandenburg Medical School, Germany (Study ID: 190032024-ANF). The findings align with the STROBE guidelines for observational studies [16].

### Study design, setting, and data sources

This cross-sectional study utilized aggregated data from all hospitalizations across Germany in 2023. Data were based on the §21 of the Hospital Remuneration Act and sourced from the Institute for the Remuneration System in the Hospital Sector (InEK GmbH, Siegburg, Germany). Data on total hospice admissions were retrieved from the Federal Health Reporting Service (Gesundheitsberichterstattung des Bundes, https://www.gbe-bund.de/). Information regarding regional hospice bed density (beds per one million inhabitants) and the density of specialized outpatient palliative care (SAPV) teams (teams per one million inhabitants) was sourced from the pallCompare Monitor (https://www.bifg.de/projekte/pallcompare). As 2023 figures were unavailable for these specific metrics, data from 2024 were utilized for the analysis. Furthermore, the proportion of deceased individuals who received SAPV care during their final year of life was also extracted from the pallCompare Monitor. For this metric, 2022 was utilized as the most recent year of available data. Finally, the at-risk-of-poverty rate and the Gini index for 2023 were retrieved from the Federal Statistical Office of Germany (Statistisches Bundesamt) via the official sustainability reporting portal (https://www.statistikportal.de/de/nachhaltigkeit).

### Cohort definition, participants, and study size

We defined the following inclusion criteria (1): classified as inpatient cases, (2) treated in 2023, and (3) involving patients aged 18 years or older. The study size reflects the total number of hospital stays meeting these criteria in 2023. We identified adult patients who were transferred from hospital to an inpatient hospice and compared this group with the overall cohort of inpatient cases in 2023. Due to technical constraints, the analysis focused on hospital cases without access to individual patient-level data.

### Definitions and variables

As part of the German DRG system, discharge reasons must be coded and reported to INEK. Discharge to an inpatient hospice is classified under code 11, “Discharge to a hospice,” in accordance with Appendix 2 to Section 301 of the German Social Code Book V. Diagnoses were categorized using the International Statistical Classification of Diseases and Related Health Problems, 10^th^ Revision, German Modification (ICD-10-GM). The DRG system assigns a single primary diagnosis per case while allowing multiple secondary diagnoses. Medical procedures and treatments were identified using the corresponding Operations and Procedures Codes (OPS) (16, 17).

The analysis examined the following variables:

- Total number of hospitalizations in 2023
- Reasons for hospital discharge or transfer, as documented in Section 301 of the German Social Code Book V
- Total number of hospital cases within each cohort
- Distribution of sex and age
- Primary and secondary diagnoses
- Hospital distribution based on bed capacity and ownership type

As the German Index of Socioeconomic Deprivation (GISD) is not available at the federal state level, alternative indicators were utilized to assess regional socioeconomic conditions. We employed the at-risk-of-poverty rate (defined as the percentage of the population falling below the state-specific median income) and the Gini index as a standardized measure of regional income and wealth inequality.

### Addressing bias

Selection bias was minimized by including all DRG-based hospital-to-hospice transfers in Germany for 2023. Patients outside the DRG system, such as foreign self-paying patients, cannot be accounted for. Additionally, due to data protection regulations, cases with an annual frequency of fewer than five were not available. However, given the large dataset of over 15,000 cases analyzed per year, this limitation is unlikely to significantly affect the findings. As our analysis is based on insurance claims data, hospital case counts do not correspond directly to individual patients, as a single patient may experience multiple hospitalizations within a given year, particularly when receiving home care. However, because hospital cases may include multiple admissions per patient, whereas hospice transfers typically occur only once per individual, the observed proportion of hospital-to-hospice transfers remains a valid representation at the case level. To mitigate measurement bias, relevant cases were identified based on designated codes, associated diagnoses, and treatments using standardized ICD-10 coding. Although billing data may introduce minor classification errors, the consistent application of coding standards reduces this risk. Nonetheless, coding omissions remain a potential issue, particularly for non-revenue-relevant DRG codes recorded as secondary diagnoses and treatments. However, this does not apply to primary diagnoses or revenue-critical codes related to surgical procedures.

### Data management and statistical analysis

Data were extracted from the InEK data browser and organized using Microsoft Excel for Mac (Version 16.93, Microsoft Corporation, Redmond, WA, USA). GraphPad Prism 9 for macOS (Version 9.5.1, GraphPad Software, La Jolla, CA, USA) was used for statistical analysis and visualization.

Descriptive statistics were used to calculate frequencies and ratios. The total number of cases for a given diagnosis included hospitalizations where the diagnosis appeared as either primary or secondary. To quantify disparities, we calculated Odds Ratios (OR) for age and gender and Risk Ratios (RR) for regional variations, including 95% confidence intervals (CI). Regional RR were calculated using the state with the lowest transfer rate (Bavaria) as the reference. CIs were estimated via log-transformation of the ratios, following standard epidemiological procedures (18). Linear regression analysis was performed to assess the association between regional hospice transfer rates (dependent variable) and inpatient hospice bed capacity per one million inhabitants (independent variable).

## Results

### German hospital cases

In 2023, German hospitals managed a total of 17,063,249 inpatient cases of which 15,200,893 inpatient cases were adult patients aged ≥18 years. Female patients accounted for 52.5% of these cases (7,977,960), male patients for 47.5% (7,222,035) and diverse patients or patients with unknown gender comprised 898 cases. Patients aged 65 years or older constituted 54.2% of all hospital cases (8,241,611, **Table 1**).

**Table 1:** Epidemiology.

	Total number of all hospital cases including patients ≥ 18 years		Fatal hospital cases including patients ≥ 18 years	
	number of cases	%	number of cases	%
All cases	15,200,893		439,315	
Gender				
Female	7,977,960	52.5%	202,203	46.0%
Male	7,222,035	47.5%	236,915	53.9%
Diverse	293	0%	1	0%
Unknown	605	0%	196	0%
Age groups				
18-29 years	1,031,366	6.8%	1,150	0.3%
30-39 years	1,334,304	8.8%	2,893	0.7%
40-49 years	1,117,669	7.4%	7,086	1.6%
50-54 years	820,652	5.4%	8,533	1.9%
55-59 years	1,209,646	8.0%	16,602	3.8%
60-64 years	1,445,645	9.5%	26,737	6.1%
65-74 years	3,089,257	20.3%	85,890	19.6%
75-79 years	1,458,679	9.6%	54,291	12.4%
80+ years	3,693,675	24.3%	236,133	53.8%
≥65 years	8,241,611	54.2%	376.314	85.7%

**Table 1** depicts an overview of case numbers, as well as age and sex distribution, among all hospitalized cases and and fatal cases in Germany in 2023.

### Hospital discharges and cohort transferred to hospice

**Figure 1:**
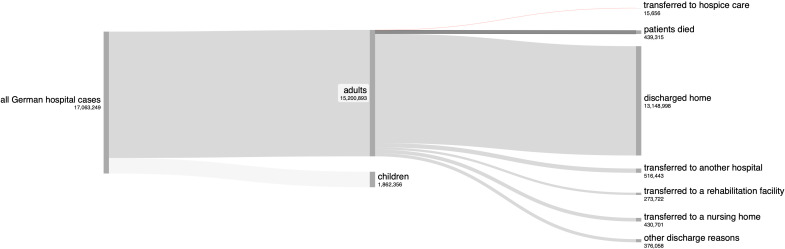
Sankey diagram illustrating discharges from German hospitals.

**Figure 2:**
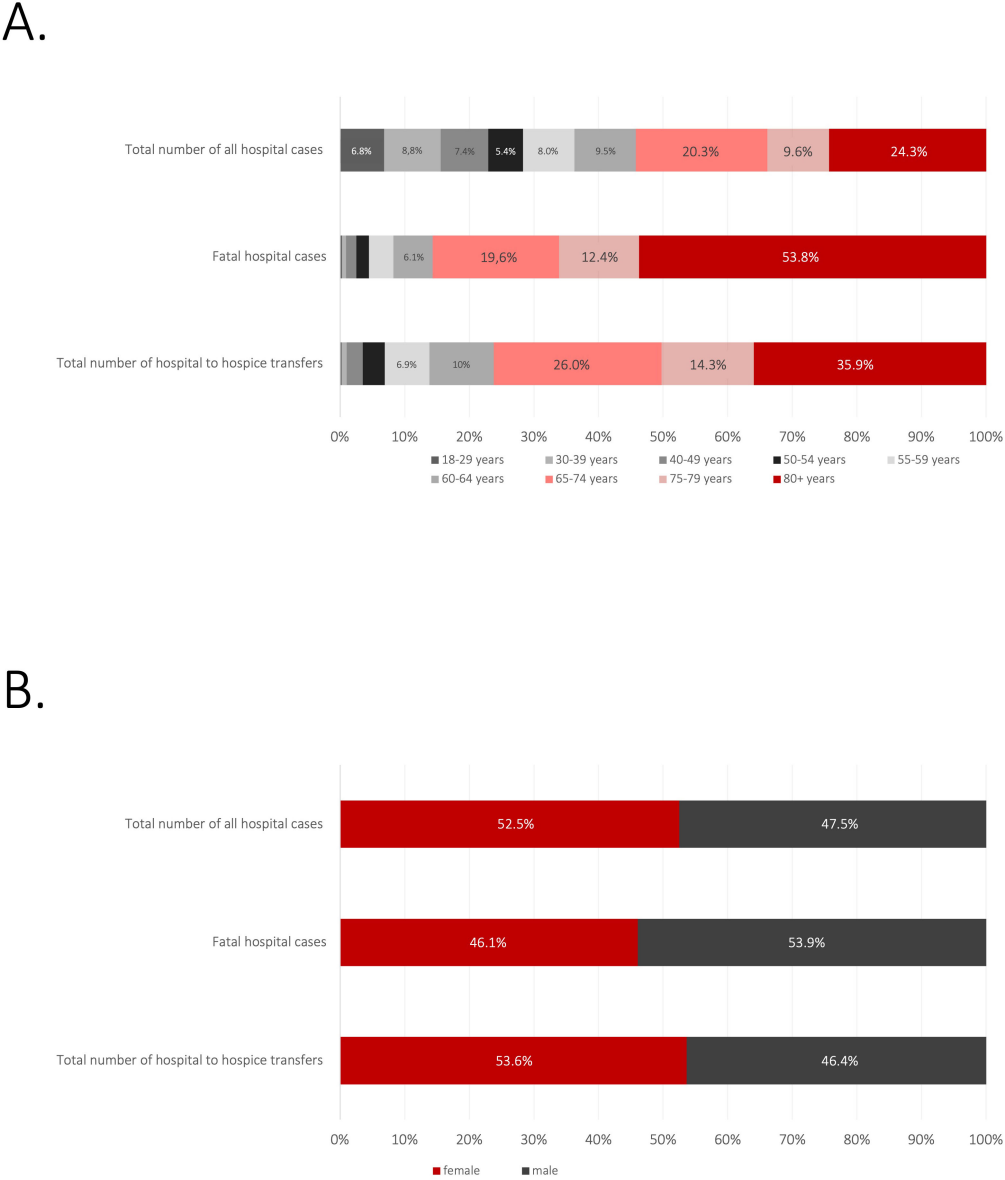
Age groups and gender distribution. **Figure 2** shows the relative age **(A)** and gender distribution **(B)** of all German hospital cases, fatal cases and those cases, in which patients were discharged to hospice care.

Among all hospital cases involving adult patients, the vast majority (13,148,998 cases, 86.5%, see **Figure 1**) resulted in patients being discharged home. A smaller proportion of patients were transferred to other facilities: 3.4% (516,443 cases) were transferred to another hospital, 1.8% (273,722 cases) to a rehabilitation facility, and 2.8% (430,701 cases) to nursing homes. Other discharge reasons accounted for 2.5% (376,058 cases). 2.9% (439,315 cases) of patients died during hospitalization. Among these, 46% were female (202,203 cases) and 54% were male (236,915 cases, with 197 cases classified as unknown or diverse). The odds of mortality were significantly lower for female patients compared to males (OR 0.77; 95% CI 0.76–0.77; Chi-square = 7093, df = 1, p <.0001; **Figure 2**).

**Figure 3:**
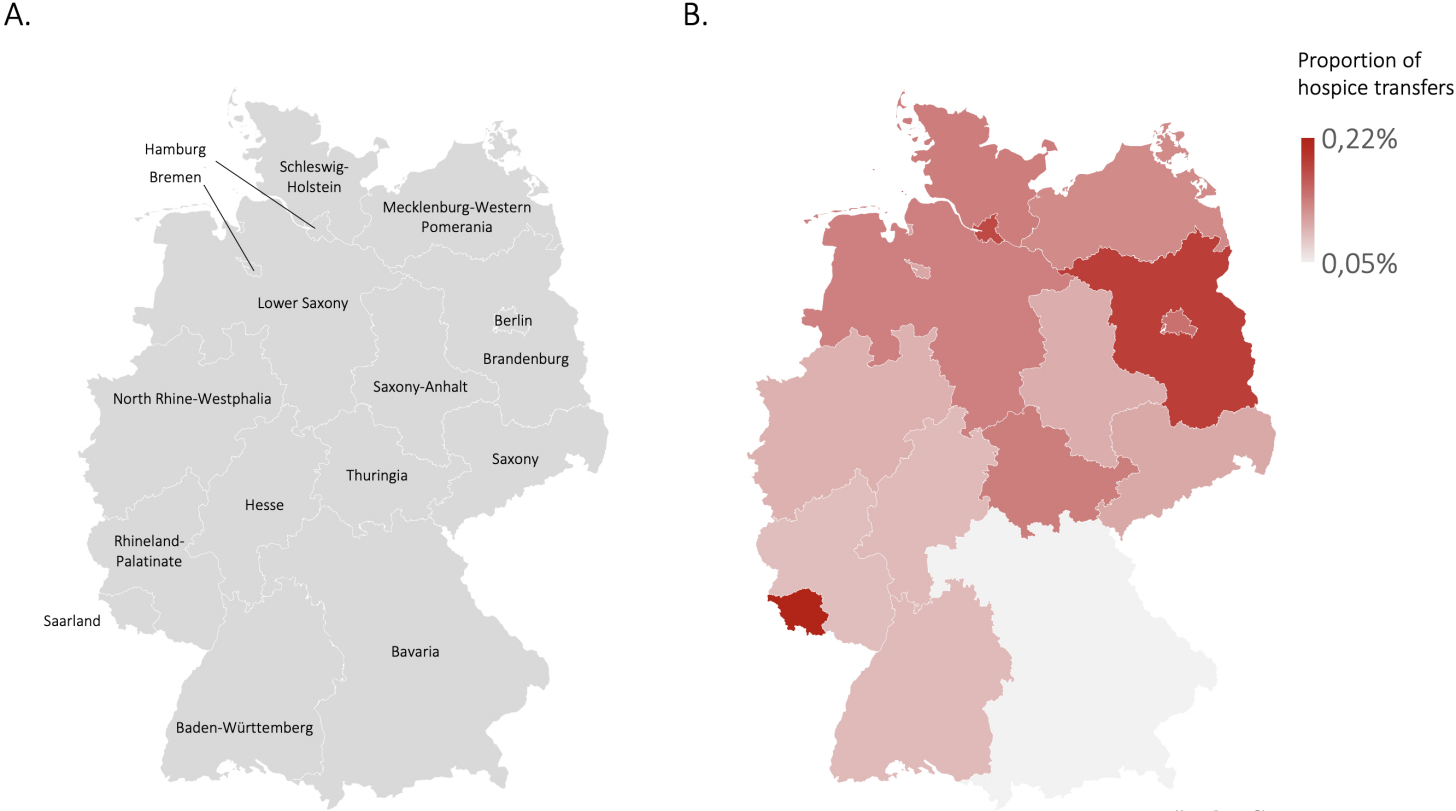
Percentage of hospital cases resulting in patient transfers to hospice. **Figure 3** illustrates the percentage of hospital cases resulting in patient transfers to hospice in each federal state of Germany **(B)**. Panel **(A)** provides a reference map of Germany indicating the federal states.

In a total of 15,656 hospital cases (0,12% of all German hospital cases), patients were transferred to inpatient hospices. However, the rate of hospice transfers varied significantly across federal states (**Figure 3**). The highest rates were observed in Saarland (0.2% of all hospital cases in Saarland) and Brandenburg (0.19%), while Bavaria had by far the lowest rate (0.05%, **Table 2**). Significant regional disparities were observed in hospital-to-hospice transfer rates across Germany, with Risk Ratios (RR) ranging from 1.00 in Bavaria (95% CI 0.92–1.08; reference) to a maximum of 4.36 in Saarland (95% CI 3.92–4.85) and Brandenburg (RR 3.70; 95% CI 3.39–4.05). These findings demonstrate that even after accounting for regional case volumes, the likelihood of a hospice transfer remains highly dependent on geographical location. Linear regression analysis revealed a significant positive correlation between the hospital-to-hospice transfer rate (2023) and regional hospice bed capacity per one million inhabitants (2024). The model demonstrates that bed capacity is a strong predictor of transfer rates (y = 229.6x + 7.9; R² = 0.65; F = 23.96; p = 0.0003). The hospital-to-hospice transfer rate showed no significant correlation with the density of specialized outpatient palliative care (SAPV) teams per one million inhabitants, the proportion of deceased individuals who received SAPV care, nor with the socioeconomic indicators (at-risk-of-poverty rate and Gini index; p > 0.05 for each). This suggests that regional transfer patterns are primarily driven by inpatient infrastructure rather than outpatient availability or socioeconomic disparities. .

**Table 2:** Hospital to hospice transfer: cases in federal states.

	Total number of all hospital cases	Total number of hospital to hospice transfers	Hospital-to-hospice transfers (%)	Hospice bed per 1 million inhabitants	Relative risk (Bavaria as reference)	Lower 95-CI	Upper 95-CI
Germany	15,200,893	15,656	0.10%		2.05	1.94	2.18
Federal states							
Baden-Württemberg	1,658,292	1,503	0.09%	22.8	1.80	1.67	1.95
Bavaria	2,352,149	1,180	0.05%	17.1	1,00	0.92	1.08
Berlin	690,722	998	0.14%	57.3	2.88	2.65	3.13
Brandenburg	431,509	802	0.19%	56.4	3.70	3.38	4.05
Bremen	166,351	173	0.10%	23.4	2.07	1.77	2.43
Hamburg	386,846	678	0.18%	44.4	3.49	3,18	3.84
Hesse	1,074,931	971	0.09%	35.2	1.80	1,66	1.96
Mecklenburg-Western Pomerania	344,664	430	0.12%	44.2	2.49	2.23	2.78
Lower Saxony	1,340,675	1,771	0.13%	35.5	2.63	2,45	2.83
North Rhine-Westphalia	3,633,091	3,517	0.10%		1.93	1.81	2.06
North Rhine				40.6			
Westphalia-Lippe				36.3			
Rhineland-Palatinate	732,164	645	0.09%	22.8	1,76	1.6	1.93
Saarland	214,916	470	0.22%	48.4	4,36	3.92	4.85
Saxony	790,355	810	0.10%	27.2	2,04	1.87	2.23
Saxony-Anhalt	440,623	436	0.10%	29.3	1,97	1.78	2.20
Schleswig-Holstein	474,554	640	0.13%	35.9	2,69	2.44	2.96
Thuringia	469,051	632	0.13%	46.5	2,69	2.44	2.96

Of the hospice transfers, 8,399 patients (53.6%) were female, and 7,257 (46.4%) were male. Compared to all adult hospital cases in Germany, the proportion of female patients in the hospice transfer cohort was slightly but significantly higher compared to the total sample (53.6% vs. 52.5%, Chi-square 8.5, df 1, p = .004). In 11,933 of the cases, patients were aged 65 or older (76.2% of all hospice transfers). The proportion of elderly patients was significantly higher in the hospice cohort compared to the cohort of all German hospital cases (Chi-square 3050, df 1, p <.0001; OR: 1,41, 95%-CI: 1,37 - 1,44). Mortality risk was substantially higher in the elderly population with patients aged 65 or older having had nearly 43 times higher odds of a fatal outcome compared to those aged 18–29 (OR 42.86; 95% CI 40.44–45.43; p < 0.001).

According to Federal Health Reporting Service (GBE), in 2023 a total of 47,846 patients received care in inpatient hospices across Germany. Therefore, approximately one-third of hospice admissions involved patients transitioning from a hospital setting.

### Diagnoses

Oncological diseases constituted the majority of primary diagnoses in hospital cases involving patient transfers to hospices. A malignant oncological disease was the primary diagnosis in 10,768 cases (68.8% of all discharges to hospice) and appeared as a secondary diagnosis in 20,980 cases. The coding system permits multiple secondary diagnoses but only one primary diagnosis, which explains this distribution. Among the most frequent primary diagnoses, malignant lung tumors accounted for 2,156 cases (13.8% of all hospice discharges), followed by intestinal tumors in 1,087 cases (6.9%), malignant pancreatic tumors in 975 cases (6.2%), and breast cancer in 804 cases (5.1%). Heart failure was the most common non-oncological primary diagnosis, recorded in 336 cases (2.2%). **Figure 3** presents an overview of diagnoses in hospital cases leading to hospice transfer.

Metastatic disease was frequently documented as a secondary diagnosis. Liver or bile duct metastases appeared in 19.4% of all hospital cases resulting in hospice transfer, bone metastases in 17.4%, lung metastases in 13%, and brain metastases in 2.6%. Tumour-related anemia occurred in 10%. Infections were also prevalent, with urinary tract infections diagnosed in 17.3% of cases, Covid-19 in 5%, Clostridium difficile-induced enterocolitis in 1.3%, and sepsis in 2.3%. Additionally, delirium was recorded as a secondary diagnosis in 7.1% of cases, hemiparesis in 7%, depressive episodes in 6.9%, and epilepsy or status epilepticus in 6.6%.

Creutzfeldt-Jakob disease had the highest percentage of hospital to hospice transfers, occurring in 10.1% of cases (28 out of 276). Brain malignancies accounted for 3.4% of hospice transfers (625 out of 18,621 cases), while pancreatic malignancies had a transfer rate of 1.6% (975 out of 59,699 cases).

**Figure 4:**
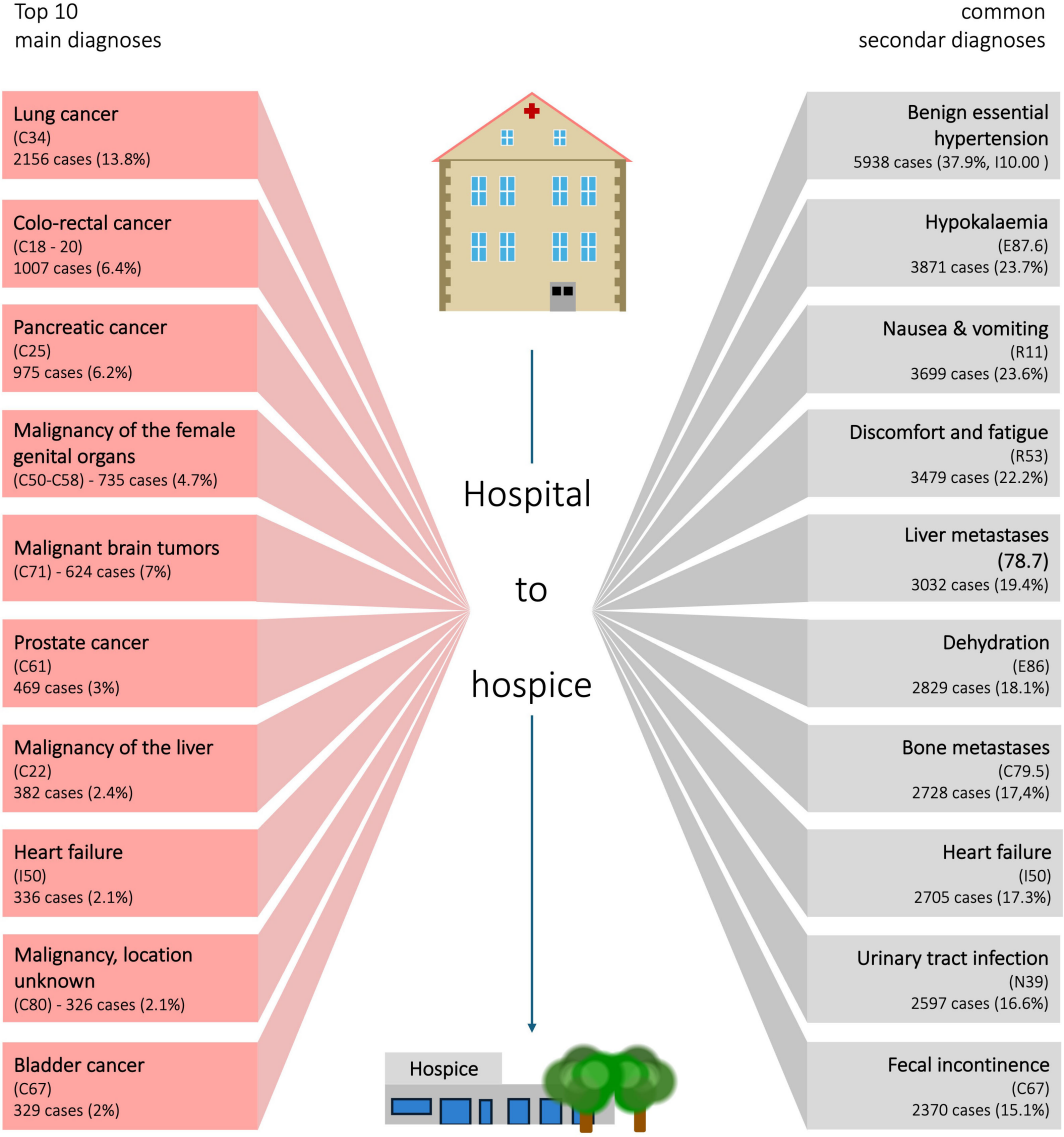
Common main and secondary diagnoses in hospital cases resulting in patient transfers to hospice.

Patients discharged to hospices had a range of significant comorbidities (**Table 3**, **Figure 4**, **Supplementary Table 1**). Among the 15,656 cases, renal failure or chronic kidney disease was coded as secondarydiagnoses in 4315 cases (27.6%), heart failure in 3135 cases (20%), diabetes mellitus in 3203 cases (20,5%) and chronic pulmonary diseases in 1726 cases (11.2%). A detailed list of relevant comorbidities, based on the Charlson Comorbidity Index, is provided in **Supplementary Table 1**.

**Table 3:** Main diagnoses and secondary diagnoses (Comorbidities).

Description	ICD-10-GM codes	Main diagnoses		Secondary diagnoses	
Total		15656	100.0%	15656	100.0%
Human immunodeficiency virus disease	B20 - 24			9	0.1%
Solid tumors	C00 - C76; C80	9647	61.6%	4551	29.1%
Metastases	C77 – 79	648	4.1%	15816	101.0%
Lymphomas/Leukemias	C81 -97	473	3.0%	251	1.6%
Diabetes mellitus	E10 - E13	14	0.1%	3203	20.5%
Dementia	F00 - 03; F05.1; G30 - 31	10	0.1%	1119	7.1%
Heart failure	I11; I13.00/.20; I50	336	2.1%	3135	20.0%
Myocardial infarction	I21	23	0.1%	87	0.6%
Cerebrovascular diseases	I61 - 66; G45 - 46	296	1.9%	546	3.5%
Peripheral arterial occlusive disease	I70 - I71	25	0.2%	658	4.2%
Chronic pulmonary disease	J44; J80 - 84	281	1.8%	1746	11.2%
Ulcer	K25 - 26			256	1.6%
Liver cirrhosis/liver failure	K70 - 74	140	0.9%	1130	7.2%
Collagenoses	M05 - 06; M32 - 35			130	0.8%
Renal failure/Chronic kidney disease	N17 - 19	111	0.7%	4315	27.6%

## Discussion

Our analysis reveals the following key findings:

- 2023, 15,656 adult patients (0.1% of all German hospital cases) were transferred from hospitals to inpatient hospices, representing roughly one-third of all hospice admissions in Germany.
- Over 75% of transfers involved patients aged 65 or older, with oncological diseases as the primary diagnosis in nearly 70% of cases.
- Hospital-to-hospice transfer rates correlate with regional hospice bed capacity but show no association with SAPV density, SAPV utilization, or socioeconomic indicators (p>0.05).

### The hospital-to-hospice interface in Germany with high regional disparities

This study provides a comprehensive epidemiological profile of the hospital-to-hospice interface in Germany. Nearly 300 inpatient hospices provide end-of-life care (293 hospices listed in the Hospice and Palliative Care Directory as of March 12, 2025) (19). In 2023, just under one-third of the 47,846 inpatient hospice cases were direct hospital transfers. The cohort predominantly comprises elderly patients where 75% are aged 65 or older and those with oncological primary diagnoses represent nearly 70% of the group. While specific data are lacking, the remaining two-thirds of admissions likely occurred via general practice physicians or specialized outpatient palliative care (SAPV) services. Overall, hospital-to-hospice transfers accounted for only 0.12% of all hospital discharges. For context, 439,315 patients (2.9% of adult cases) died in hospitals during the same period. We observed marked regional disparities in hospital to hospice transfers. Patients treated in high-capacity federal states such as Saarland and Brandenburg had more than a fourfold higher likelihood of transfer than patients in Bavaria. These findings align with an analysis of BARMER health insurance data from 2016 to 2019, which reported that 3.3% of deceased individuals received inpatient hospice care (20, 21). That study likewise identified substantial regional variation, with Bremen and Bavaria reporting the lowest utilization rates at 1.6% and 2.0%, respectively, and Berlin and Hamburg reporting the highest at 5.6% and 5.4% (20, 21).

### Structural determinism and supply-dependent care

Hospital-to-hospice transfer rates correlate strongly with regional hospice bed capacity. Conversely, SAPV density, its utilization, and socioeconomic indicators show no significant association. This pattern suggests a paradigm of supply-sensitive care where transfers depend primarily on regional hospice capacity rather than outpatient palliative care availability or socioeconomic deprivation. Similar findings emerged from the PallCompare project and the analysis of SAPV provision in Germany. That research demonstrated substantial regional variation in palliative care utilization that could not be explained by patient characteristics or objective medical need according to reference (21, 22). Instead, regional care capacity, provider incentives within differing reimbursement systems, and historically developed care cultures accounted for much of the observed variation as noted in reference (21, 22). Socioeconomic deprivation does not appear to influence access to or provision of inpatient palliative care, even following hospital structural reforms, such as those implemented in Germany’s largest federal state, North Rhine–Westphalia (23).

### The “referral gap”: international benchmarks

Comparison with international benchmarks suggests a systemic referral gap in Germany. In the Netherlands and the United States, hospitals serve as the primary referral source for hospice care and account for approximately 52% of admissions (24, 25). In contrast, the German rate of 32.7% indicates structural constraints in discharge management following acute hospital treatment. This comparatively low proportion likely reflects capacity driven care and limited hospice bed availability.

Our findings also diverge from patterns observed in England and the United States, where socioeconomic disadvantage frequently constitutes a major barrier to hospice access (26, 27). In Germany, regional infrastructure appears to outweigh individual socioeconomic status in determining access. These results point to a distinctive structural feature of the German system. Access to hospice care depends less on personal financial resources and more on geographical location and regional service capacity.

### Demographic vulnerability: aging and gender as systemic indicators

The data confirm the critical role of hospice care for the geriatric population. Older patients more frequently present with oncological diseases and multiple comorbidities (28, 29). These individuals often endure a high symptom burden across physical, psychological, and social domains at the end of life within various care settings (28–34). Furthermore, older patients consistently prefer palliative care that prioritizes quality of life, symptom relief, and support for caregivers rather than life prolongation or aggressive interventions (35–37). These preferences remain dynamic and change over time as health status, prognosis, and personal values evolve (36, 38). Such shifts underscore the necessity for person-centered advance care planning that adapts to the patient’s journey. Despite these clear preferences, older patients often experience lower quality of care and poorer symptom control than younger cohorts as noted in reference. This discrepancy highlights a significant gap between patient needs and the cur. rent delivery of geriatric end-of-life care (30–34).

Significant gender differences emerged as women transitioned to hospices more frequently despite their lower overall hospital mortality. This disparity likely reflects the collapse of informal support systems. Because women often outlive their partners, they frequently lack the spousal care networks necessary for home-based palliative care. Demographic data confirm that widowed women constitute the majority of elderly care recipients and often live alone (39, 40). This social isolation increases reliance on institutional settings as children or extended social networks provide less stable support than spousal care (40). The structure of the German palliative care system further drives these transitions. Limited outpatient capacity and regional variability often force women to move to hospices or assisted living earlier when informal support proves insufficient (39, 40). As demographic changes increase demand while personnel resources decrease, available regional capacities rather than individual needs often determine the form of care provided. This supply-sensitive logic disproportionately affects elderly women without spousal caregivers (20, 21).

### Policy implications and strategic healthcare planning

This study demonstrates that the hospital-to-hospice interface in Germany follows a supply-driven logic where regional capacity outweighs clinical demand. Expert healthcare planning must recognize that expanding outpatient services like SAPV does not automatically substitute for inpatient beds as SAPV density showed no correlation with transfer rates. As German healthcare policy moves toward a closer integration of the inpatient and outpatient sectors, these findings provide crucial insights into the structural barriers that currently hinder a seamless patient journey at the end of life. Consequently, health authorities must critically evaluate whether existing hospice capacities sufficiently meet the actual clinical demand or if current transfer rates merely reflect available space. To ensure equitable access, policymakers must address these structural determinisms by focusing on both infrastructure and the underlying care cultures that influence clinical decision-making.

Our findings align with the theoretical access model of Penchansky and Thomas which identifies five dimensions of healthcare entry: availability, accessibility, acceptability, affordability, and accommodation (23, 41). This analysis specifically highlights accessibility as the primary barrier to hospice care. Future development should prioritize patient-centered networks that integrate digital solutions and telehealth to bridge the gap between acute hospitals and hospices (42–45). Implementing real-time capacity management and digital consultation tools can transition the system from a capacity-based to a needs-based model. By fostering interdisciplinary digital platforms, Germany can ensure that a dignified end-of-life transition remains a universal right rather than a geographic coincidence.

### Limitations

This study has several limitations:

1. The number of hospital cases does not directly correspond to individual patients, as patients may experience multiple hospitalizations within a year, particularly when receiving home care. This means a single patient could theoretically have multiple transfers from a hospital to a hospice. However, hospices typically focus solely on quality-of-life-centered therapy, so transfers from a hospice to a hospital are generally not part of their model of care. Based on our case-based approach, a higher proportion of patients will require transfer to hospice care. Since a hospice transfer usually occurs only once per patient, the proportion of hospital-to-hospice transfers among all hospice admissions is accurately represented in our data.
2. Hospital mortality was calculated based on hospital cases, but specific causes of death were not specified.
3. Due to the unavailability of 2023 figures for certain metrics, data from 2022 and 2024 were utilized for specific variables, which potentially limits temporal comparability across all indicators, although significant structural changes in these parameters are unlikely to have occurred within this short timeframe.
4. We only analyzed data on hospital transfers to hospices, which represent roughly one-third of all hospice admissions. Data on admissions from home and from patients treated with SAPV (specialized palliative home specialized care services) are not available. Therefore, our data only represents a part of inpatient hospice - admissions in Germany.
5. The findings’ reliability depends on accurate and consistent coding practices in the InEK database. Misclassification or inconsistent coding could introduce biases or inaccuracies. However, reasons for discharge and primary diagnoses must be coded.
6. Data provided do not cover key aspects of palliative care, such as symptom burden, quality-of-life, and other patient-reported outcome measures (PROMs) and -experienced measures (PREMs) by patients and their families. Thus, the actual assessment of palliative care needs and quality of palliative care is impossible.
7. The current analysis lacks data regarding the longitudinal patient journey and the continuity of care before hospital admission which prevents a definitive assessment of whether these hospitalizations represent avoidable systemic gaps in the outpatient palliative network.
8. While this study utilizes the framework of Penchansky and Thomas, the analysis focuses exclusively on the dimension of availability and cannot account for the remaining dimensions of acceptability, affordability, or accommodation (41).

## Conclusion

Hospital-to-hospice transfers in Germany remain significantly lower than international averages. This study demonstrates that regional bed density emerges as the most influential factor in these transitions, suggesting a system driven by availability rather than individual clinical necessity. While elderly patients and women constitute the largest groups among these transitions, their frequent transfer to institutional hospice care likely reflects a broader lack of informal spousal support networks within these demographics.

As healthcare policy increasingly seeks to integrate inpatient and outpatient sectors, these findings serve as a critical benchmark for the development of integrated palliative care models. To achieve equitable healthcare, future research must evaluate whether current regional capacities are sufficient and how local care cultures dictate clinical paths. Moving from a capacity-driven infrastructure toward a needs-oriented system is vital to ensure that access to hospice care becomes a standardized right regardless of a patient’s geographic location.

## Data Availability

The original contributions presented in the study are included in the article/**Supplementary Material**. Further inquiries can be directed to the corresponding author.

## Supplementary material

Supplementary Table 1 Extended overview over comorbidities of patients transferred from hospital to hospice care The table shows mortality rates of all coded primary or secondary diagnosis.

## References

[1] Anhang Price R Tolpadi A Schlang D Bradley MA Parast L Teno JM . Characteristics of hospices providing high-quality care. J Palliat Med. (2020) 23:1639–43. doi: 10.1089/jpm.2019.0505. PMID: 32155376

[2] Breyre AM Wang DH Brooten JK Colwell CB Hanson KC Taigman M . Ems care of adult hospice patients- a position statement and resource document of Naemsp and Aahpm. Prehosp Emerg Care. (2023) 27:560–5. doi: 10.1080/10903127.2023.2193978. PMID: 36961936

[3] Towns K Dougherty E Kevork N Wiljer D Seccareccia D Rodin G . Availability of services in Ontario hospices and hospitals providing inpatient palliative care. J Palliat Med. (2012) 15:527–34. doi: 10.1089/jpm.2011.0453. PMID: 22512831

[4] Williams AM Crooks VA Whitfield K Kelley M-L Richards J-L DeMiglio L . Tracking the evolution of hospice palliative care in Canada: A comparative case study analysis of seven provinces. BMC Health Serv Res. (2010) 10:147. doi: 10.1186/1472-6963-10-147. PMID: 20515491 PMC2898768

[5] Klinger CA Howell D Zakus D Deber RB . Barriers and facilitators to care for the terminally ill: A cross-country case comparison study of Canada, England, Germany, and the United States. Palliat Med. (2014) 28:111–20. doi: 10.1177/0269216313493342. PMID: 23801462

[6] Panarella M Saarela O Esensoy AV Jakda A Liu ZA . Regional variation in palliative care receipt in Ontario, Canada. J Palliat Med. (2019) 22:1370–7. doi: 10.1089/jpm.2018.0573. PMID: 31090480

[7] Finlay IG . Developing a template to plan palliative care services: The Welsh experience. J Pain Symptom Manage. (2009) 38:81–6. doi: 10.1016/j.jpainsymman.2009.04.018. PMID: 19615631

[8] Alt-Epping B Nauck F . Spezialisierte ambulante palliativversorgung (Sapv). Bundesgesundheitsbl Gesundheitsforsch Gesundheitsschutz. (2015) 58:430–5. doi: 10.1007/s00103-015-2125-6. PMID: 25673015

[9] Melching H . Neue gesetzliche regelungen für die palliativversorgung und ihre implikationen für politik und praxis. Bundesgesundheitsbl Gesundheitsforsch Gesundheitsschutz. (2017) 60:4–10. doi: 10.1007/s00103-016-2480-y. PMID: 27995269

[10] Kamp MA Willert A-C Dunkl V Kowski AB Golla H . Palliative begleitung bei neuroonkologischen erkrankungen: Schwerkranke menschen in multidisziplinären teams behandeln. DNP–Die Neurol Psychiatr. (2025) 26:35–42. doi: 10.1007/s15202-025-6389-x. PMID: 41770356

[11] GKV-SpitzenverbandArbeiterwohlfahrt Bundesverband e. V.Bundesverband Kinderhospiz e. V.Deutscher Caritasverband e. V.Deutscher Hospiz- und PalliativVerband e. V.Deutscher Kinderhospizverein e. V . Rahmenvereinbarung nach § 39a Abs. 1 Satz 4 Sgb V über Art und Umfang sowie Sicherstellung der stationären Hospizversorgung in der Fassung vom 18.11.2024. In: GKV-Spitzenverband (2024). Berlin: GKV Spitzenverband. Available online at: https://www.gkv-spitzenverband.de/media/dokumente/krankenversicherung_1/hospiz_palliativversorgung/2024-11-18_Rahmenvereinbarung_39a_Abs1_Satz_4:SGB_V_stat_Hospizversorgung_Erw.pdf. (Accessed March 12, 2026).

[12] Izumi S Noble BN Candrian CB Tjia J Bordley J Mensik J . Health care worker perceptions of gaps and opportunities to improve hospital-to-hospice transitions. J Palliat Med. (2020) 23:900–6. doi: 10.1089/jpm.2019.0513. PMID: 31895623

[13] Peuten S Jaspers B Hainsch-Muller I Aulmann C Schneider W Radbruch L . Concept-dependent and -independent care effects of site-specific care concepts using "Pain" as an example. Schmerz. (2024) 38:433–40. doi: 10.1007/s00482-023-00754-1. PMID: 37773298 PMC11576803

[14] Kasdorf A Dust G Vennedey V Rietz C Polidori MC Voltz R . What are the risk factors for avoidable transitions in the last year of life? A qualitative exploration of professionals' perspectives for improving care in Germany. BMC Health Serv Res. (2021) 21:147. doi: 10.1186/s12913-021-06138-4. PMID: 33588851 PMC7885553

[15] Wang SY Aldridge MD Gross CP Canavan M Cherlin E Johnson-Hurzeler R . Transitions between healthcare settings of hospice enrollees at the end of life. J Am Geriatr Soc. (2016) 64:314–22. doi: 10.1111/jgs.13939. PMID: 26889841 PMC4762182

[16] Bundesinstitut für Arzneimittel und Medizinprodukte . Internationale statistische klassifikation der Krankheiten und verwandter Gesundheitsprobleme, 10. Revision, German Modification, Version 2024. In: Bundesinstitut für Arzneimittel und Medizinprodukte (2025). Berlin: Bundesinstitut für Arzneimittel und Medizinprodukte. Available online at: https://klassifikationen.bfarm.de/icd-10-gm/kode-suche/htmlgm2024/index.htm. (Accessed March 12, 2026).

[17] Bundesinstitut für Arzneimittel und Medizinprodukte . Operationen- und prozedurenschlüssel, Version 2024 (2025). Available online at: https://klassifikationen.bfarm.de/ops/kode-suche/htmlops2024/index.htm. (Accessed March 12, 2026).

[18] Altman DG . Practical Statistics for Medical Research. New York: Taylor & Francis (1990).

[19] Deutsche Gesellschaft für Palliativmedizin . Wegweiser Hospiz- und Palliativversorgung in Deutschland (2025). Available online at: https://wegweiser-hospiz-palliativmedizin.de. (Accessed March 12, 2026)

[20] Ditscheid B Krause M Lehmann T Stichling K Jansky M Nauck F . Palliative care at the end of life in Germany: Utilization and regional distribution. Bundesgesundheitsbl Gesundheitsforsch Gesundheitsschutz. (2020) 63:1502–10. doi: 10.1007/s00103-020-03240-6. PMID: 33185710 PMC7686196

[21] Ditscheid B Meissner F Gebel C Hennig B Marschall U Meissner W . Utilization of palliative care at the end of life in Germany: Temporal trend (2016-2019) and regional variability. Bundesgesundheitsbl Gesundheitsforsch Gesundheitsschutz. (2023) 66:432–42. doi: 10.1007/s00103-023-03683-7. PMID: 36897332 PMC10063517

[22] Freytag A Meissner F Krause M Lehmann T Jansky MK Marschall U . A regional comparison of outcomes quality and costs of general and specialized palliative care in Germany: A claims data analysis. Bundesgesundheitsbl Gesundheitsforsch Gesundheitsschutz. (2023) 66:1135–45. doi: 10.1007/s00103-023-03746-9. PMID: 37535086 PMC10539464

[23] Von Sass C Wedding U Bergmann J Fink L Adelstein J Van Oorschot B . Impact of the hospital structural reform and socio-economic deprivation in North Rhine-Westphalia (Nrw) on the accessibility of specialized inpatient palliative care. Gesundheitswesen. (2025). doi: 10.1055/a-2633-5943. PMID: 40997822

[24] West E Pasman HR Galesloot C Lokker ME Onwuteaka-Philipsen B Euro I . Hospice care in the Netherlands: Who applies and who is admitted to inpatient care? BMC Health Serv Res. (2016) 16:33. doi: 10.1186/s12913-016-1273-1. PMID: 26821859 PMC4730778

[25] Furuno JP Noble BN McPherson ML Lapane KL Sera L Izumi S . Variation in hospice patient and admission characteristics by referral location. Med Care. (2020) 58:1069–74. doi: 10.1097/mlr.0000000000001415. PMID: 32925461

[26] Sleeman KE De Brito M Etkind S Nkhoma K Guo P Higginson IJ . The escalating global burden of serious health-related suffering: Projections to 2060 by world regions, age groups, and health conditions. Lancet Glob Health. (2019) 7:e883–92. doi: 10.1016/S2214-109X(19)30172-X. PMID: 31129125 PMC6560023

[27] Barclay JS Kuchibhatla M Tulsky JA Johnson KS . Association of hospice patients' income and care level with place of death. JAMA Intern Med. (2013) 173:450–6. doi: 10.1001/jamainternmed.2013.2773. PMID: 23420383 PMC3889123

[28] Grudzen CR Richardson LD Morrison M Cho E Morrison RS . Palliative care needs of seriously ill, older adults presenting to the emergency department. Acad Emerg Med. (2010) 17:1253–7. doi: 10.1111/j.1553-2712.2010.00907.x. PMID: 21175525 PMC3058630

[29] Ryan T Ingleton C Gardiner C Parker C Gott M Noble B . Symptom burden, palliative care need and predictors of physical and psychological discomfort in two UK hospitals. BMC Palliat Care. (2013) 12:11. doi: 10.1186/1472-684X-12-11. PMID: 23442926 PMC3599055

[30] Lindskog M Tavelin B Lundstrom S . Old age as risk indicator for poor end-of-life care quality - a population-based study of cancer deaths from the Swedish Register of Palliative Care. Eur J Cancer. (2015) 51:1331–9. doi: 10.1016/j.ejca.2015.04.001. PMID: 25958036

[31] Lindemann K Martinsson L Kaasa S Lindquist D . Elderly gynaecological cancer patients at risk for poor end of life care: A population-based study from the Swedish Register of Palliative Care. Acta Oncol. (2020) 59:636–43. doi: 10.1080/0284186X.2020.1744717. PMID: 32238040

[32] Quinn KL Shurrab M Gitau K Kavalieratos D Isenberg SR Stall NM . Association of receipt of palliative care interventions with health care use, quality of life, and symptom burden among adults with chronic noncancer illness: A systematic review and meta-analysis. JAMA. (2020) 324:1439–50. doi: 10.1001/jama.2020.14205. PMID: 33048152 PMC8094426

[33] Grudzen CR Siman N Cuthel AM Adeyemi O Yamarik RL Goldfeld KS . Palliative care initiated in the emergency department: A cluster randomized clinical trial. JAMA. (2025) 333:599–608. doi: 10.1001/jama.2024.23696. PMID: 39813042 PMC11836764

[34] Morita T Kuriya M Miyashita M Sato K Eguchi K Akechi T . Symptom burden and achievement of good death of elderly cancer patients. J Palliat Med. (2014) 17:887–93. doi: 10.1089/jpm.2013.0625. PMID: 25083586

[35] Waller A Sanson-Fisher R Nair BR Evans T . Preferences for end-of-life care and decision making among older and seriously ill inpatients: A cross-sectional study. J Pain Symptom Manage. (2020) 59:187–96. doi: 10.1016/j.jpainsymman.2019.09.003. PMID: 31539600

[36] Gonzalez-Gonzalez AI Schmucker C Nothacker J Nury E Dinh TS Brueckle MS . End-of-life care preferences of older patients with multimorbidity: A mixed methods systematic review. J Clin Med. (2020) 10. doi: 10.3390/jcm10010091. PMID: 33383951 PMC7795676

[37] Johnston BM Daveson B Normand C Ryan K Smith M McQuillan R . Preferences of older people with a life-limiting illness: A discrete choice experiment. J Pain Symptom Manage. (2022) 64:137–45. doi: 10.1016/j.jpainsymman.2022.04.180. PMID: 35490993

[38] Robinson L Dewhurst F Huggin A Stow D Stenson C Westhead E . Exploring older people's end-of-life care preferences over time: A scoping review. Palliat Med. (2025) 39:665–77. doi: 10.1177/02692163251331161. PMID: 40285379 PMC12102515

[39] Dasch B Lenz P . Der sterbeort von tumorpatienten im zeitlichen trend. Der Onkologe. (2021) 27:602–9. doi: 10.1007/s00761-021-00926-7. PMID: 41770356

[40] Dorin L Krupa E Metzing S Buscher A . Gender disparities in German home-care arrangements. Scand J Car Sci. (2016) 30:164–74. doi: 10.1111/scs.12236. PMID: 26036651

[41] Penchansky R Thomas JW . The concept of access: Definition and relationship to consumer satisfaction. Med Care. (1981) 19:127–40. doi: 10.1097/00005650-198102000-00001. PMID: 7206846

[42] Greer JA Jacobs J Pensak N MacDonald JJ Fuh CX Perez GK . Randomized trial of a tailored cognitive-behavioral therapy mobile application for anxiety in patients with incurable cancer. Oncologist. (2019) 24:1111–20. doi: 10.1634/theoncologist.2018-0536. PMID: 30683710 PMC6693695

[43] Greer JA Jacobs JM Pensak N Nisotel LE Fishbein JN MacDonald JJ . Randomized trial of a smartphone mobile app to improve symptoms and adherence to oral therapy for cancer. J Natl Compr Canc Netw. (2020) 18:133–41. 32023526 10.6004/jnccn.2019.7354

[44] Greer JA Temel JS El-Jawahri A Rinaldi S Kamdar M Park ER . Telehealth vs in-person early palliative care for patients with advanced lung cancer: A multisite randomized clinical trial. JAMA. (2024) 332:1153–64. doi: 10.1001/jama.2024.13964. PMID: 39259563 PMC11391365

[45] May S Gehlhaar A Stahlhut K Kamp MA Heinze M Allsop M . Let's put it this way: You can't really live without it - Digital technologies in routine palliative care delivery: An explorative qualitative study with patients and their family caregivers in Germany. BMC Health Serv Res. (2024) 24:702. doi: 10.1186/s12913-024-11150-5. PMID: 38831314 PMC11149286

