# Supplementary material for: Hospital-to-hospice transfers in Germany: analyzing the impact of age and gender at the care transition interface
Source: Front Oncol. 2026 Mar 26;16:1743124. doi: 10.3389/fonc.2026.1743124 (PMC13061869; doi:10.3389/fonc.2026.1743124)
Supplement: Supplementary Table 1 — Extended overview over comorbidities of patients transferred from hospital to hospice care The table shows mortality rates of all coded primary or secondary diagnosis. [file Table1.docx]

| **ICD-10-GM codes** | **Description** | **Main diagnoses** | | **Secondary diagnoses** | |
| --- | --- | --- | --- | --- | --- |
|  |  |  |  |  |  |
| **Total** |  | **15656** | **100,0%** | **15656** | **100,0%** |
|  |  |  |  |  |  |
| **Myocardial infarction** | | **23** | **0,1%** | **87** | **0,6%** |
| I21.0 | Acute anterior wall transmural myocardial infarction | 7 | 0,0% | 5 | 0,0% |
| I21.1 | Acute posterior wall transmural myocardial infarction |  |  | 5 | 0,0% |
| I21.4 | Acute subendocardial myocardial infarction | 16 | 0,1% | 71 | 0,5% |
| I21.9 | Acute myocardial infarction; unspecified |  |  | 6 | 0,0% |
|  |  |  |  |  |  |
| **Heart failure** |  | **336** | **2,1%** | **3135** | **20,0%** |
| I11.00 | Hypertensive heart disease with (congestive) heart failure: Without indication of a hypertensive crisis |  |  | 341 | 2,2% |
| I11.01 | Hypertensive heart disease with (congestive) heart failure: With indication of a hypertensive crisis |  |  | 25 | 0,2% |
| I13.00 | Hypertensive heart and kidney disease with (congestive) heart failure: Without indication of a hypertensive crisis |  |  | 13 | 0,1% |
| I13.20 | Hypertensive heart and kidney disease with (congestive) heart failure and kidney failure: Without indication of a hypertensive crisis |  |  | 51 | 0,3% |
| I50.00 | Primary right heart failure |  |  | 20 | 0,1% |
| I50.01 | Secondary right heart failure | 136 | 0,9% | 799 | 5,1% |
| I50.02 | Right heart failure without symptoms |  |  | 9 | 0,1% |
| I50.03 | Right heart failure with symptoms during greater exertion |  |  | 34 | 0,2% |
| I50.04 | Right heart failure with symptoms during lighter exertion |  |  | 53 | 0,3% |
| I50.05 | Right heart failure with symptoms at rest |  |  | 58 | 0,4% |
| I50.11 | Left heart failure: Without symptoms |  |  | 64 | 0,4% |
| I50.12 | Left heart failure: With symptoms during greater exertion | 7 | 0,0% | 358 | 2,3% |
| I50.13 | Left heart failure: With symptoms during lighter exertion | 53 | 0,3% | 571 | 3,6% |
| I50.14 | Left heart failure: With symptoms at rest | 129 | 0,8% | 516 | 3,3% |
| I50.19 | Left heart failure: Unspecified | 5 | 0,0% | 92 | 0,6% |
| I50.9 | Heart failure; unspecified | 6 | 0,0% | 131 | 0,8% |
|  |  |  |  |  |  |
| **Peripheral arterial occlusive disease** | | **25** | **0,2%** | **658** | **4,2%** |
| I70.20 | Atherosclerosis of the extremity arteries: pelvic-leg type; without symptoms |  |  | 47 | 0,3% |
| I70.21 | Atherosclerosis of the extremity arteries: pelvic-leg type; with exercise-induced ischemic pain; walking distance 200 m and more |  |  | 42 | 0,3% |
| I70.22 | Atherosclerosis of the extremity arteries: pelvic-leg type; with exercise-induced ischemic pain; walking distance less than 200 m |  |  | 121 | 0,8% |
| I70.23 | Atherosclerosis of the extremity arteries: pelvic-leg type; with resting pain |  |  | 38 | 0,2% |
| I70.24 | Atherosclerosis of the extremity arteries: pelvic-leg type; with ulceration |  |  | 72 | 0,5% |
| I70.25 | Atherosclerosis of the extremity arteries: pelvic-leg type; with gangrene | 25 | 0,2% | 97 | 0,6% |
| I70.29 | Atherosclerosis of the extremity arteries: other and unspecified |  |  | 160 | 1,0% |
| I70.8 | Atherosclerosis of other arteries |  |  | 13 | 0,1% |
| I70.9 | Generalized and unspecified atherosclerosis |  |  | 11 | 0,1% |
| I71.2 | Aneurysma der Aorta thoracica; ohne Angabe einer Ruptur |  |  | 13 | 0,1% |
| I71.4 | Aneurysma der Aorta abdominalis; ohne Angabe einer Ruptur |  |  | 44 | 0,3% |
|  |  |  |  |  |  |
| **Cerebrovascular diseases** | | **296** | **1,9%** | **546** | **3,5%** |
| I61.0 | Intracerebral hemorrhage in the cerebral hemisphere; subcortical | 28 | 0,2% | 26 | 0,2% |
| I61.1 | Intracerebral hemorrhage in the cerebral hemisphere; cortical |  |  | 9 | 0,1% |
| I61.2 | Intracerebral hemorrhage in the cerebral hemisphere; unspecified |  |  | 10 | 0,1% |
| I61.5 | Intracerebral intraventricular hemorrhage | 5 | 0,0% | 9 | 0,1% |
| I61.6 | Intracerebral hemorrhage at multiple locations | 8 | 0,1% | 6 | 0,0% |
| I61.8 | Other intracerebral hemorrhage |  |  | 27 | 0,2% |
| I61.9 | Intracerebral hemorrhage; unspecified | 5 | 0,0% | 15 | 0,1% |
| I62.00 | Nontraumatic subdural hemorrhage: acute |  |  | 8 | 0,1% |
| I62.01 | Nontraumatic subdural hemorrhage: subacute |  |  | 7 | 0,0% |
| I62.02 | Nontraumatic subdural hemorrhage: chronic |  |  | 15 | 0,1% |
| I62.09 | Nontraumatic subdural hemorrhage: unspecified |  |  | 5 | 0,0% |
| I62.1 | Nontraumatic extradural hemorrhage |  |  |  | 0,0% |
| I62.9 | Intracranial hemorrhage (nontraumatic); unspecified |  |  | 5 | 0,0% |
| G45.12 | Internal carotid artery syndrome (hemiplegic): complete regression within 1 to 24 hours |  |  |  | 0,0% |
| G45.89 | Other cerebral transient ischemia and related syndromes: course of regression unspecified |  |  |  | 0,0% |
| G45.92 | Cerebral transient ischemia; unspecified: complete regression within 1 to 24 hours |  |  |  | 0,0% |
| G46.0 | Middle cerebral artery syndrome |  |  | 50 | 0,3% |
| G46.2 | Posterior cerebral artery syndrome |  |  | 7 | 0,0% |
| G46.3 | Brain stem syndrome |  |  |  | 0,0% |
| G46.4 | Cerebellar syndrome |  |  | 6 | 0,0% |
| G46.8 | Other syndromes of the cerebral vessels in cerebrovascular diseases |  |  | 21 | 0,1% |
| I63.0 | Cerebral infarction due to thrombosis of precerebral arteries | 9 | 0,1% |  | 0,0% |
| I63.1 | Cerebral infarction due to embolism of precerebral arteries | 8 | 0,1% | 9 | 0,1% |
| I63.2 | Cerebral infarction due to unspecified occlusion or stenosis of precerebral arteries | 6 | 0,0% | 5 | 0,0% |
| I63.3 | Cerebral infarction due to thrombosis of cerebral arteries | 29 | 0,2% | 26 | 0,2% |
| I63.4 | Cerebral infarction due to embolism of cerebral arteries | 112 | 0,7% | 57 | 0,4% |
| I63.5 | Cerebral infarction due to unspecified occlusion or stenosis of cerebral arteries | 51 | 0,3% | 46 | 0,3% |
| I63.8 | Other cerebral infarction | 24 | 0,2% | 41 | 0,3% |
| I63.9 | Cerebral infarction; unspecified referred to | 11 | 0,1% | 26 | 0,2% |
| I64 | Stroke; not referred to as hemorrhage or infarction |  |  | 5 | 0,0% |
| I65.0 | Occlusion and stenosis of the vertebral artery |  |  | 7 | 0,0% |
| I65.1 | Occlusion and stenosis of the basilar artery |  |  |  | 0,0% |
| I65.2 | Occlusion and stenosis of the carotid artery |  |  | 86 | 0,5% |
| I65.3 | Occlusion and stenosis of multiple and bilateral precerebral arteries |  |  | 7 | 0,0% |
| I66.0 | Occlusion and stenosis of the middle cerebral artery |  |  |  | 0,0% |
| I66.9 | Occlusion and stenosis of an unspecified cerebral artery |  |  | 5 | 0,0% |
|  |  |  |  |  |  |
| **Dementia** |  | **10** | **0,1%** | **1119** | **7,1%** |
| F00.0 | Dementia in Alzheimer's disease; early onset (type 2) |  |  | 53 | 0,3% |
| F00.1 | Dementia in Alzheimer's disease; late onset (type 1) |  |  | 24 | 0,2% |
| F00.2 | Dementia in Alzheimer's disease; atypical or mixed form |  |  | 19 | 0,1% |
| F00.9 | Dementia in Alzheimer's disease; unspecified |  |  | 9 | 0,1% |
| F01.1 | Multi-infarct dementia |  |  | 12 | 0,1% |
| F01.2 | Subcortical vascular dementia |  |  | 16 | 0,1% |
| F01.3 | Mixed cortical and subcortical vascular dementia |  |  | 62 | 0,4% |
| F01.8 | Other vascular dementia |  |  | 28 | 0,2% |
| F01.9 | Vascular dementia; unspecified |  |  | 16 | 0,1% |
| F02.3 | Dementia in primary Parkinson's syndrome |  |  | 27 | 0,2% |
| F02.8 | Dementia in conditions classified elsewhere |  |  | 6 | 0,0% |
| F03 | Unspecified dementia |  |  | 575 | 3,7% |
| F05.1 | Delirium in dementia | 10 | 0,1% | 144 | 0,9% |
| G30.0 | Early onset Alzheimer's disease |  |  |  | 0,0% |
| G30.1 | Late onset Alzheimer's disease |  |  | 53 | 0,3% |
| G30.8 | Other Alzheimer's disease |  |  | 22 | 0,1% |
| G30.9 | Alzheimer's disease; unspecified |  |  | 18 | 0,1% |
| G31.0 | Circumscribed brain atrophy |  |  | 23 | 0,1% |
| G31.1 | Senile degeneration of the brain; not elsewhere classified |  |  | 6 | 0,0% |
| G31.2 | Alcoholic degeneration of the nervous system |  |  |  | 0,0% |
| G31.9 | Degenerative disease of the nervous system; unspecified |  |  | 6 | 0,0% |
|  |  |  |  |  |  |
| **Chronic pulmonary disease** | | **281** | **1,8%** | **1746** | **11,2%** |
| J44.00 | Chronic obstructive pulmonary disease with acute lower respiratory tract infection: FEV1 <35% of predicted | 44 | 0,3% | 41 | 0,3% |
| J44.01 | Chronic obstructive pulmonary disease with acute lower respiratory tract infection: FEV1 >=35% and <50% of predicted | 6 | 0,0% | 34 | 0,2% |
| J44.02 | Chronic obstructive pulmonary disease with acute lower respiratory tract infection: FEV1 >=50% and <70% of predicted |  |  | 17 | 0,1% |
| J44.03 | Chronic obstructive pulmonary disease with acute lower respiratory tract infection: FEV1 >=70% of predicted |  |  | 5 | 0,0% |
| J44.09 | Chronic obstructive pulmonary disease with acute lower respiratory tract infection: FEV1 unspecified | 34 | 0,2% | 125 | 0,8% |
| J44.10 | Chronic obstructive pulmonary disease with acute exacerbation; unspecified: FEV1 <35% of predicted | 73 | 0,5% | 59 | 0,4% |
| J44.11 | Chronic obstructive pulmonary disease with acute exacerbation; unspecified: FEV1 >=35% and <50% of predicted | 9 | 0,1% | 15 | 0,1% |
| J44.12 | Chronic Obstructive pulmonary disease with acute exacerbation; unspecified: FEV1 >=50% and <70% of predicted |  |  | 14 | 0,1% |
| J44.13 | Chronic obstructive pulmonary disease with acute exacerbation; unspecified: FEV1 >=70% of predicted |  |  | 5 | 0,0% |
| J44.19 | Chronic obstructive pulmonary disease with acute exacerbation; unspecified: FEV1 unspecified | 62 | 0,4% | 107 | 0,7% |
| J44.80 | Other specified chronic obstructive pulmonary disease: FEV1 <35% of predicted | 15 | 0,1% | 47 | 0,3% |
| J44.81 | Other specified chronic obstructive pulmonary disease: FEV1 >=35% and <50% of predicted |  |  | 57 | 0,4% |
| J44.82 | Other specified chronic obstructive pulmonary disease: FEV1 >=50% and <70% of predicted |  |  | 70 | 0,4% |
| J44.83 | Other specified chronic obstructive pulmonary disease: FEV1 >=70% of predicted |  |  | 32 | 0,2% |
| J44.89 | Other specified chronic obstructive pulmonary disease: FEV1 unspecified designated |  |  | 453 | 2,9% |
| J44.90 | Chronic obstructive pulmonary disease; unspecified: FEV1 <35% of predicted | 6 | 0,0% | 26 | 0,2% |
| J44.91 | Chronic obstructive pulmonary disease; unspecified: FEV1 >=35% and <50% of predicted |  |  | 16 | 0,1% |
| J44.92 | Chronic obstructive pulmonary disease; unspecified: FEV1 >=50% and <70% of predicted |  |  | 19 | 0,1% |
| J44.93 | Chronic obstructive pulmonary disease; unspecified: FEV1 >=70% of predicted |  |  | 12 | 0,1% |
| J44.99 | Chronic obstructive pulmonary disease; unspecified: FEV1 unspecified | 6 | 0,0% | 317 | 2,0% |
| J80.02 | Adult respiratory distress syndrome [ARDS]: Moderate adult respiratory distress syndrome [ARDS] |  |  |  | 0,0% |
| J80.03 | Adult respiratory distress syndrome [ARDS]: Severe adult respiratory distress syndrome [ARDS] |  |  | 5 | 0,0% |
| J80.09 | Adult respiratory distress syndrome [ARDS]: Adult respiratory distress syndrome [ARDS]; severity unspecified |  |  |  | 0,0% |
| J45.09 | Predominantly allergic bronchial asthma |  |  | 20 | 0,1% |
| J45.19 | Non-allergic asthma: Control status and severity not specified |  |  | 10 | 0,1% |
| J45.89 | Mixed asthma: Control status and severity not specified |  |  | 14 | 0,1% |
| J45.90 | Asthma; unspecified: Reported as well controlled and not severe |  |  | 17 | 0,1% |
| J45.99 | Asthma; unspecified: Control status and severity not specified |  |  | 130 | 0,8% |
| J80.03 | Adult respiratory distress syndrome [ARDS]: Severe adult respiratory distress syndrome [ARDS] |  |  | 5 | 0,0% |
| J81 | Pulmonary edema |  |  | 16 | 0,1% |
| J84.10 | Other interstitial lung diseases with fibrosis: No acute exacerbation specified | 10 | 0,1% | 41 | 0,3% |
| J84.11 | Other interstitial lung diseases with fibrosis: With acute exacerbation specified | 16 | 0,1% | 12 | 0,1% |
| J84.90 | Interstitial lung disease; unspecified: No acute exacerbation specified |  |  | 5 | 0,0% |
|  |  |  |  |  |  |
|  |  |  |  |  |  |
| **Collagenoses** |  | **0** | **0,0%** | **130** | **0,8%** |
| M35.3 | Polymyalgia rheumatica |  |  | 18 | 0,1% |
| M32.1 | Systemic lupus erythematosus with involvement of organs or organ systems |  |  |  | 0,0% |
| M32.9 | Systemic lupus erythematosus; unspecified |  |  |  | 0,0% |
| M05.30 | Seropositive chronic polyarthritis with involvement of other organs and organ systems: Multiple locations |  |  |  | 0,0% |
| M05.39 | Seropositive chronic polyarthritis with involvement of other organs and organ systems: Unspecified location |  |  |  | 0,0% |
| M05.80 | Other seropositive chronic polyarthritis: Multiple locations |  |  |  | 0,0% |
| M05.90 | Seropositive chronic polyarthritis; unspecified: Multiple locations |  |  |  | 0,0% |
| M05.99 | Seropositive chronic polyarthritis; unspecified: Unspecified location |  |  |  | 0,0% |
| M06.00 | Seronegative chronic polyarthritis: Multiple locations |  |  | 6 | 0,0% |
| M06.80 | Other specified chronic polyarthritis: Multiple locations |  |  | 10 | 0,1% |
| M06.89 | Other specified chronic polyarthritis: Unspecified location |  |  | 5 | 0,0% |
| M06.90 | Chronic polyarthritis; unspecified: Multiple locations |  |  | 40 | 0,3% |
| M06.98 | Chronic polyarthritis; unspecified: Other [neck; head; ribs; Trunk; skull; spine] |  |  |  | 0,0% |
| M06.99 | Chronic polyarthritis; unspecified: Unspecified location |  |  | 51 | 0,3% |
| M33.1 | Other dermatomyositis |  |  |  | 0,0% |
|  |  |  |  |  |  |
| **Ulcer** |  | **0** | **0,0%** | **256** | **1,6%** |
| K25.0 | Gastric ulcer: Acute; with bleeding |  |  | 42 | 0,3% |
| K25.1 | Gastric ulcer: Acute; with perforation |  |  |  | 0,0% |
| K25.3 | Gastric ulcer: Acute; without bleeding or perforation |  |  | 65 | 0,4% |
| K25.4 | Gastric ulcer: Chronic or unspecified; with bleeding |  |  | 10 | 0,1% |
| K25.7 | Gastric ulcer: Chronic; without bleeding or perforation |  |  |  | 0,0% |
| K25.9 | Gastric ulcer: Neither acute nor chronic; without bleeding or perforation |  |  | 16 | 0,1% |
| K26.0 | Duodenal ulcer: Acute; with bleeding |  |  | 58 | 0,4% |
| K26.1 | Duodenal ulcer: Acute; with perforation |  |  |  | 0,0% |
| K26.3 | Duodenal ulcer: Acute; without bleeding or perforation |  |  | 44 | 0,3% |
| K26.4 | Duodenal ulcer: Chronic or unspecified; with bleeding |  |  | 5 | 0,0% |
| K26.7 | Duodenal ulcer: Chronic; without bleeding or perforation |  |  | 6 | 0,0% |
| K26.9 | Duodenal ulcer: Neither acute nor chronic; without bleeding or perforation |  |  | 10 | 0,1% |
|  |  |  |  |  |  |
| **Liver cirrhosis / liver failure** | | **140** | **0,9%** | **1130** | **7,2%** |
| K70.3 | Alcoholic liver cirrhosis | 80 | 0,5% | 179 | 1,1% |
| K70.40 | Acute and subacute alcoholic liver failure |  |  | 8 | 0,1% |
| K70.41 | Chronic alcoholic liver failure |  |  | 5 | 0,0% |
| K70.42 | Acute-on-chronic alcoholic liver failure | 5 | 0,0% |  | 0,0% |
| K71.0 | Toxic liver disease with cholestasis |  |  | 29 | 0,2% |
| K71.9 | Toxic liver disease; unspecified |  |  | 9 | 0,1% |
| K72.0 | Acute and subacute liver failure; not elsewhere classified | 7 | 0,0% | 111 | 0,7% |
| K72.10 | Acute-on-chronic liver failure; not elsewhere classified |  |  | 8 | 0,1% |
| K72.18 | Other and unspecified chronic liver failure; not elsewhere classified |  |  | 22 | 0,1% |
| K72.71 | Hepatic encephalopathy grade 1 |  |  | 31 | 0,2% |
| K72.72 | Hepatic encephalopathy grade 2 |  |  | 57 | 0,4% |
| K72.73 | Hepatic encephalopathy grade 3 |  |  | 42 | 0,3% |
| K72.79 | Hepatic encephalopathy grade unspecified |  |  | 34 | 0,2% |
| K72.9 | Liver failure; unspecified |  |  | 49 | 0,3% |
| K74.0 | Liver fibrosis |  |  | 10 | 0,1% |
| K74.6 | Other and unspecified liver cirrhosis | 48 | 0,3% | 234 | 1,5% |
| K74.70 | Liver cirrhosis; Child-Pugh stage A |  |  | 55 | 0,4% |
| K74.71 | Liver cirrhosis; Child-Pugh stage B |  |  | 107 | 0,7% |
| K74.72 | Liver cirrhosis; Child-Pugh stage C |  |  | 140 | 0,9% |
|  |  |  |  |  |  |
| **Diabetes mellitus** |  | **14** | **0,1%** | **3203** | **20,5%** |
| E10.11 | Type 1 diabetes mellitus: With ketoacidosis: Described as derailed |  |  | 5 |  |
| E10.90 | Type 1 diabetes mellitus: Without complications: Not described as derailed |  |  | 17 |  |
| E10.91 | Type 1 diabetes mellitus: Without complications: Described as derailed |  |  | 17 |  |
| E11.20 | Type 2 diabetes mellitus: With renal complications: Not described as derailed |  |  | 154 |  |
| E11.21 | Type 2 diabetes mellitus: With renal complications: Described as derailed |  |  | 54 |  |
| E11.30 | Type 2 diabetes mellitus: With ocular complications: Not described as derailed |  |  | 6 |  |
| E11.40 | Type 2 diabetes mellitus: With neurological complications: Not described as derailed |  |  | 65 |  |
| E11.41 | Type 2 diabetes mellitus: With neurological complications: Described as derailed |  |  | 22 |  |
| E11.50 | Type 2 diabetes mellitus: With peripheral vascular complications: Not described as derailed |  |  | 24 |  |
| E11.51 | Type 2 diabetes mellitus: With peripheral vascular complications: Described as derailed designated |  |  | 8 |  |
| E11.60 | Diabetes mellitus; type 2: With other specified complications: Not designated as derailed |  |  | 36 |  |
| E11.61 | Diabetes mellitus; type 2: With other specified complications: Designated as derailed |  |  | 41 |  |
| E11.72 | Diabetes mellitus; type 2: With multiple complications: With other multiple complications; not designated as derailed |  |  | 75 |  |
| E11.73 | Diabetes mellitus; type 2: With multiple complications: With other multiple complications; designated as derailed |  |  | 45 |  |
| E11.74 | Diabetes mellitus; type 2: With multiple complications: With diabetic foot ulcer; not designated as derailed | 7 | 0,0% | 63 | 0,4% |
| E11.75 | Diabetes mellitus; type 2: With multiple complications: With diabetic foot ulcer; designated as derailed |  |  | 19 |  |
| E11.90 | Diabetes mellitus; type 2: Without complications: Not designated as derailed |  |  | 1939 |  |
| E11.91 | Diabetes mellitus; type 2: Without complications: Designated as derailed | 7 | 0,0% | 474 | 3,0% |
| E13.21 | Other specified diabetes mellitus: With renal complications: Designated as Described as derailed |  |  | 5 |  |
| E13.90 | Other specified diabetes mellitus: Without complications: Not described as derailed |  |  | 84 |  |
| E13.91 | Other specified diabetes mellitus: Without complications: Described as derailed |  |  | 50 |  |
|  |  |  |  |  |  |
| **Renal failure / Chronic kidney disease** | | **111** | **0,7%** | **4315** | **27,6%** |
| N17.81 | Sonstiges akutes Nierenversagen: Stadium 1 |  |  | 12 |  |
| N17.82 | Sonstiges akutes Nierenversagen: Stadium 2 |  |  | 14 |  |
| N17.83 | Sonstiges akutes Nierenversagen: Stadium 3 |  |  | 19 |  |
| N17.91 | Akutes Nierenversagen; nicht näher bezeichnet: Stadium 1 | 5 | 0,0% | 434 | 2,8% |
| N17.92 | Akutes Nierenversagen; nicht näher bezeichnet: Stadium 2 | 9 | 0,1% | 554 | 3,5% |
| N17.93 | Akutes Nierenversagen; nicht näher bezeichnet: Stadium 3 | 68 | 0,4% | 570 | 3,6% |
| N17.99 | Akutes Nierenversagen; nicht näher bezeichnet: Stadium nicht näher bezeichnet | 5 | 0,0% | 71 | 0,5% |
| N18.1 | Chronische Nierenkrankheit; Stadium 1 |  |  | 9 |  |
| N18.2 | Chronische Nierenkrankheit; Stadium 2 |  |  | 310 |  |
| N18.3 | Chronische Nierenkrankheit; Stadium 3 |  |  | 1412 |  |
| N18.4 | Chronische Nierenkrankheit; Stadium 4 |  |  | 489 |  |
| N18.5 | Chronische Nierenkrankheit; Stadium 5 | 24 | 0,2% | 148 | 0,9% |
| N18.89 | Sonstige chronische Nierenkrankheit; Stadium nicht näher bezeichnet |  |  | 5 |  |
| N18.9 | Chronische Nierenkrankheit; nicht näher bezeichnet |  |  | 37 |  |
| N19 | Nicht näher bezeichnete Niereninsuffizienz |  |  | 231 |  |
| N18.9 | Chronic kidney disease; unspecified |  |  |  |  |
| N19 | Unspecified renal failure |  |  |  |  |
|  |  |  |  |  |  |
| **Human immunodeficiency virus disease** | | **0** | **0,0%** | **9** | **0,1%** |
| B24 | Unspecified HIV disease [Human immunodeficiency virus disease] |  |  | 9 |  |
|  |  |  |  |  |  |
| **Solid tumors** |  | **9647** | **61,6%** | **4551** | **29,1%** |
|  |  |  |  |  |  |
| **Metastases** |  | **648** | **4,1%** | **15816** | **101,0%** |
|  |  |  |  |  |  |
| **Lymphomas/Leukemias** | | **473** | **3,0%** | **251** | **1,6%** |
|  |  |  |  |  |  |
| C01 | Malignant neoplasm of the base of the tongue | 16 | 0,1% | 11 | 0,1% |
| C02.1 | Malignant neoplasm: Margin of the tongue | 15 | 0,1% | 7 | 0,0% |
| C02.8 | Malignant neoplasm: Tongue; overlapping multiple parts | 12 | 0,1% | 6 | 0,0% |
| C04.0 | Malignant neoplasm: Anterior part of the floor of the mouth | 5 | 0,0% |  | 0,0% |
| C04.1 | Malignant neoplasm: Lateral part of the floor of the mouth | 6 | 0,0% |  | 0,0% |
| C04.8 | Malignant neoplasm: Floor of the mouth; overlapping multiple parts | 16 | 0,1% | 7 | 0,0% |
| C04.9 | Malignant neoplasm: Floor of the mouth; unspecified | 15 | 0,1% | 8 | 0,1% |
| C06.8 | Malignant neoplasm: Other and unspecified parts of the mouth; overlapping multiple parts | 7 | 0,0% |  | 0,0% |
| C06.9 | Malignant neoplasm: Mouth; unspecified | 5 | 0,0% |  | 0,0% |
| C07 | Malignant neoplasm of the parotid gland | 10 | 0,1% |  | 0,0% |
| C09.8 | Malignant neoplasm: Tonsil; overlapping multiple parts | 6 | 0,0% |  | 0,0% |
| C09.9 | Malignant neoplasm: Tonsil; unspecified | 5 | 0,0% |  | 0,0% |
| C10.8 | Malignant neoplasm: Oropharynx; overlapping multiple parts | 49 | 0,3% | 16 | 0,1% |
| C10.9 | Malignant neoplasm: Oropharynx; unspecified | 23 | 0,1% | 14 | 0,1% |
| C11.8 | Malignant neoplasm: Nasopharynx; overlapping multiple parts | 6 | 0,0% |  | 0,0% |
| C13.8 | Malignant neoplasm: Hypopharynx; multiple parts Overlapping | 25 | 0,2% | 12 | 0,1% |
| C13.9 | Malignant neoplasm: Hypopharynx; unspecified | 17 | 0,1% | 8 | 0,1% |
| C15.0 | Malignant neoplasm: Cervical esophagus | 5 | 0,0% |  | 0,0% |
| C15.1 | Malignant neoplasm: Thoracic esophagus | 13 | 0,1% | 12 | 0,1% |
| C15.2 | Malignant neoplasm: Abdominal esophagus | 8 | 0,1% |  | 0,0% |
| C15.3 | Malignant neoplasm: Esophagus; upper third | 21 | 0,1% | 10 | 0,1% |
| C15.4 | Malignant neoplasm: Esophagus; middle third | 25 | 0,2% | 8 | 0,1% |
| C15.5 | Malignant neoplasm: Esophagus; lower third | 80 | 0,5% | 41 | 0,3% |
| C15.8 | Malignant neoplasm: Esophagus; multiple overlapping segments | 31 | 0,2% | 11 | 0,1% |
| C15.9 | Malignant neoplasm: Esophagus; unspecified | 34 | 0,2% | 27 | 0,2% |
| C16.0 | Malignant neoplasm: Cardia | 134 | 0,9% | 44 | 0,3% |
| C16.1 | Malignant neoplasm: Fundus of the stomach | 8 | 0,1% |  | 0,0% |
| C16.2 | Malignant neoplasm: Corpus of the stomach | 71 | 0,5% | 29 | 0,2% |
| C16.3 | Malignant neoplasm: Pyloric antrum | 55 | 0,4% | 28 | 0,2% |
| C16.4 | Malignant neoplasm: Pylorus | 10 | 0,1% |  | 0,0% |
| C16.5 | Malignant neoplasm: Lesser curvature of the stomach; unspecified | 6 | 0,0% |  | 0,0% |
| C16.8 | Malignant neoplasm: Stomach; multiple overlapping segments | 59 | 0,4% | 17 | 0,1% |
| C16.9 | Malignant neoplasm: Stomach; unspecified designated | 57 | 0,4% | 30 | 0,2% |
| C17.0 | Malignant neoplasm: Duodenum | 17 | 0,1% | 16 | 0,1% |
| C17.1 | Malignant neoplasm: Jejunum | 6 | 0,0% |  | 0,0% |
| C17.2 | Malignant neoplasm: Ileum | 8 | 0,1% | 10 | 0,1% |
| C18.0 | Malignant neoplasm: Cecum | 123 | 0,8% | 53 | 0,3% |
| C18.1 | Malignant neoplasm: Vermiform appendix | 20 | 0,1% | 13 | 0,1% |
| C18.2 | Malignant neoplasm: Ascending colon | 82 | 0,5% | 57 | 0,4% |
| C18.3 | Malignant neoplasm: Right hepatic flexure of the coli | 24 | 0,2% | 18 | 0,1% |
| C18.4 | Malignant neoplasm: Transverse colon | 35 | 0,2% | 22 | 0,1% |
| C18.5 | Malignant neoplasm: Left splenic flexure of the coli | 13 | 0,1% | 13 | 0,1% |
| C18.6 | Malignant neoplasm: Descending colon | 28 | 0,2% | 18 | 0,1% |
| C18.7 | Malignant neoplasm: Sigmoid colon | 163 | 1,0% | 77 | 0,5% |
| C18.8 | Malignant neoplasm: Colon; multiple overlapping segments | 20 | 0,1% | 13 | 0,1% |
| C18.9 | Malignant neoplasm: Colon; unspecified | 53 | 0,3% | 40 | 0,3% |
| C19 | Malignant neoplasm at the rectosigmoid junction | 47 | 0,3% | 13 | 0,1% |
| C20 | Malignant neoplasm of the rectum | 399 | 2,5% | 169 | 1,1% |
| C21.0 | Malignant neoplasm: Anus; unspecified | 14 | 0,1% | 5 | 0,0% |
| C21.1 | Malignant neoplasm: Anal canal | 30 | 0,2% | 12 | 0,1% |
| C21.8 | Malignant neoplasm: Rectum; anus; and anal canal; multiple segments Overlapping | 5 | 0,0% |  | 0,0% |
| C22.0 | Hepatocellular carcinoma | 174 | 1,1% | 77 | 0,5% |
| C22.1 | Intrahepatic bile duct carcinoma | 187 | 1,2% | 58 | 0,4% |
| C22.7 | Other specified carcinomas of the liver | 8 | 0,1% | 6 | 0,0% |
| C22.9 | Malignant neoplasm: Liver; unspecified | 13 | 0,1% | 7 | 0,0% |
| C23 | Malignant neoplasm of the gallbladder | 52 | 0,3% | 19 | 0,1% |
| C24.0 | Malignant neoplasm: Extrahepatic bile duct | 87 | 0,6% | 37 | 0,2% |
| C24.1 | Malignant neoplasm: Ampulla hepatopancreatica [Ampulla of Vater] | 14 | 0,1% | 8 | 0,1% |
| C24.8 | Malignant neoplasm: Bile ducts; multiple overlapping sections | 7 | 0,0% |  | 0,0% |
| C25.0 | Malignant neoplasm: Head of pancreas | 530 | 3,4% | 172 | 1,1% |
| C25.1 | Malignant neoplasm: Body of pancreas | 98 | 0,6% | 37 | 0,2% |
| C25.2 | Malignant neoplasm: Tail of pancreas | 129 | 0,8% | 51 | 0,3% |
| C25.4 | Malignant neoplasm: Endocrine gland portion of the pancreas | 6 | 0,0% |  | 0,0% |
| C25.7 | Malignant neoplasm: Other parts of the pancreas | 7 | 0,0% | 6 | 0,0% |
| C25.8 | Malignant neoplasm: Pancreas; multiple overlapping sections | 79 | 0,5% | 19 | 0,1% |
| C25.9 | Malignant neoplasm: Pancreas; unspecified | 126 | 0,8% | 53 | 0,3% |
| C26.9 | Malignant neoplasm: Vaguely specified sites of the digestive system | 9 | 0,1% |  | 0,0% |
| C30.0 | Malignant neoplasm: Nasal cavity | 8 | 0,1% | 5 | 0,0% |
| C31.8 | Malignant neoplasm: Paranasal sinuses; multiple overlapping areas |  | 0,0% | 5 | 0,0% |
| C32.0 | Malignant neoplasm: Glottis | 7 | 0,0% |  | 0,0% |
| C32.1 | Malignant neoplasm: Supraglottis | 6 | 0,0% | 7 | 0,0% |
| C32.8 | Malignant neoplasm: Larynx; multiple overlapping areas | 16 | 0,1% | 9 | 0,1% |
| C32.9 | Malignant neoplasm: Larynx; unspecified | 10 | 0,1% | 11 | 0,1% |
| C34.0 | Malignant neoplasm: Main bronchus | 294 | 1,9% | 107 | 0,7% |
| C34.1 | Malignant neoplasm: Upper lobe (bronchus) | 770 | 4,9% | 320 | 2,0% |
| C34.2 | Malignant neoplasm: Middle lobe (bronchus) | 78 | 0,5% | 25 | 0,2% |
| C34.3 | Malignant neoplasm: Lower lobe (bronchus) | 427 | 2,7% | 148 | 0,9% |
| C34.8 | Malignant neoplasm: Bronchus and lung; multiple overlapping areas | 234 | 1,5% | 95 | 0,6% |
| C34.9 | Malignant neoplasm: Bronchus or lung; unspecified | 353 | 2,3% | 217 | 1,4% |
| C37 | Malignant neoplasm of thymus | 6 | 0,0% |  | 0,0% |
| C38.4 | Malignant neoplasm: Pleura |  | 0,0% | 8 | 0,1% |
| C40.2 | Malignant neoplasm of bone and articular cartilage: Long bones of the lower extremity | 6 | 0,0% |  | 0,0% |
| C43.3 | Malignant melanoma of other and unspecified parts of the face | 14 | 0,1% | 9 | 0,1% |
| C43.5 | Malignant melanoma of the trunk | 38 | 0,2% | 23 | 0,1% |
| C43.6 | Malignant melanoma of the upper extremity; including shoulder | 20 | 0,1% | 14 | 0,1% |
| C43.7 | Malignant melanoma of the lower extremity; including hip | 34 | 0,2% | 20 | 0,1% |
| C43.8 | Malignant melanoma of the skin; overlapping multiple sites | 5 | 0,0% |  | 0,0% |
| C43.9 | Malignant melanoma of the skin; unspecified | 33 | 0,2% | 37 | 0,2% |
| C44.2 | Other malignant neoplasms: Skin of the ear and external auditory canal | 12 | 0,1% | 7 | 0,0% |
| C44.3 | Other malignant neoplasms: Skin of other and unspecified parts of the face | 15 | 0,1% | 15 | 0,1% |
| C44.4 | Other malignant neoplasms: Scalp and neck skin | 10 | 0,1% | 8 | 0,1% |
| C44.6 | Other malignant neoplasms: Skin of the upper extremity; including shoulder |  | 0,0% | 5 | 0,0% |
| C44.7 | Other malignant neoplasms: Skin of the lower extremity; including hip |  | 0,0% | 9 | 0,1% |
| C44.8 | Other malignant neoplasms: Skin; overlapping multiple sites | 5 | 0,0% | 5 | 0,0% |
| C44.9 | Malignant neoplasm of the skin; unspecified |  | 0,0% | 8 | 0,1% |
| C45.0 | Pleural mesothelioma | 42 | 0,3% | 12 | 0,1% |
| C48.0 | Malignant neoplasm: Retroperitoneum | 11 | 0,1% |  | 0,0% |
| C48.1 | Malignant neoplasm: Specified parts of the peritoneum | 5 | 0,0% | 14 | 0,1% |
| C48.2 | Malignant neoplasm: Peritoneum; unspecified | 11 | 0,1% | 8 | 0,1% |
| C49.0 | Malignant neoplasm: Connective and other soft tissues of the head; neck Face and neck | 6 | 0,0% |  | 0,0% |
| C49.1 | Malignant neoplasm: Connective tissue and other soft tissue of the upper extremity; including the shoulder | 9 | 0,1% | 5 | 0,0% |
| C49.2 | Malignant neoplasm: Connective tissue and other soft tissue of the lower extremity; including the hip | 24 | 0,2% |  | 0,0% |
| C49.3 | Malignant neoplasm: Connective tissue and other soft tissue of the thorax | 13 | 0,1% |  | 0,0% |
| C49.4 | Malignant neoplasm: Connective tissue and other soft tissue of the abdomen | 11 | 0,1% | 10 | 0,1% |
| C49.5 | Malignant neoplasm: Connective tissue and other soft tissue of the pelvis | 9 | 0,1% | 7 | 0,0% |
| C49.8 | Malignant neoplasm: Connective tissue and other soft tissue; overlapping multiple sub-areas | 5 | 0,0% |  | 0,0% |
| C49.9 | Malignant neoplasm: Connective tissue and other soft tissue; unspecified | 15 | 0,1% | 8 | 0,1% |
| C50.1 | Malignant neoplasm: Central glandular body of the breast | 27 | 0,2% | 13 | 0,1% |
| C50.2 | Malignant neoplasm: Upper inner quadrant of the breast | 24 | 0,2% | 18 | 0,1% |
| C50.3 | Malignant neoplasm: Lower inner quadrant of the breast | 18 | 0,1% | 11 | 0,1% |
| C50.4 | Malignant neoplasm: Upper outer quadrant of the breast | 100 | 0,6% | 48 | 0,3% |
| C50.5 | Malignant neoplasm: Lower outer quadrant of the breast | 17 | 0,1% | 11 | 0,1% |
| C50.8 | Malignant neoplasm: Breast; multiple overlapping areas | 170 | 1,1% | 75 | 0,5% |
| C50.9 | Malignant neoplasm: Breast; unspecified | 448 | 2,9% | 318 | 2,0% |
| C51.0 | Malignant neoplasm of vulva: Labium majus | 5 | 0,0% |  | 0,0% |
| C51.8 | Malignant neoplasm: Vulva; multiple overlapping areas | 46 | 0,3% | 18 | 0,1% |
| C51.9 | Malignant neoplasm: Vulva; unspecified | 29 | 0,2% | 12 | 0,1% |
| C52 | Malignant neoplasm of vagina | 14 | 0,1% |  | 0,0% |
| C53.0 | Malignant neoplasm: Endocervix | 16 | 0,1% | 6 | 0,0% |
| C53.1 | Malignant neoplasm: Ectocervix | 7 | 0,0% |  | 0,0% |
| C53.8 | Malignant neoplasm: Cervix uteri; multiple overlapping areas | 45 | 0,3% | 23 | 0,1% |
| C53.9 | Malignant neoplasm: Cervix uteri; unspecified | 43 | 0,3% | 22 | 0,1% |
| C54.1 | Malignant neoplasm: Endometrium | 96 | 0,6% | 51 | 0,3% |
| C54.8 | Malignant neoplasm: Corpus uteri; multiple overlapping areas | 14 | 0,1% |  | 0,0% |
| C54.9 | Malignant neoplasm: Corpus uteri; unspecified | 12 | 0,1% | 6 | 0,0% |
| C55 | Malignant neoplasm of uterus; part unspecified | 21 | 0,1% | 13 | 0,1% |
| C56 | Malignant neoplasm of ovary | 354 | 2,3% | 137 | 0,9% |
| C57.0 | Malignant neoplasm: Falloppio's fallopian tube | 10 | 0,1% | 11 | 0,1% |
| C57.8 | Malignant neoplasm: Female genital organs multiple overlapping areas | 23 | 0,1% | 16 | 0,1% |
| C60.8 | Malignant neoplasm: penis; multiple overlapping areas | 7 | 0,0% |  | 0,0% |
| C60.9 | Malignant neoplasm: penis; unspecified |  | 0,0% | 8 | 0,1% |
| C61 | Malignant neoplasm of the prostate | 469 | 3,0% | 322 | 2,1% |
| C64 | Malignant neoplasm of the kidney; excluding renal pelvis | 157 | 1,0% | 106 | 0,7% |
| C65 | Malignant neoplasm of the renal pelvis | 35 | 0,2% | 20 | 0,1% |
| C66 | Malignant neoplasm of the ureter | 11 | 0,1% | 8 | 0,1% |
| C67.0 | Malignant neoplasm: trigone vesical wall | 8 | 0,1% | 6 | 0,0% |
| C67.2 | Malignant neoplasm: lateral bladder wall | 24 | 0,2% | 22 | 0,1% |
| C67.4 | Malignant neoplasm: posterior bladder wall | 8 | 0,1% | 9 | 0,1% |
| C67.5 | Malignant neoplasm: bladder neck | 5 | 0,0% |  | 0,0% |
| C67.6 | Malignant neoplasm: ureteral ostium | 5 | 0,0% |  | 0,0% |
| C67.8 | Malignant neoplasm: bladder; multiple overlapping areas | 183 | 1,2% | 98 | 0,6% |
| C67.9 | Malignant neoplasm: bladder; unspecified | 87 | 0,6% | 73 | 0,5% |
| C68.0 | Malignant neoplasm: urethra | 12 | 0,1% | 10 | 0,1% |
| C68.8 | Malignant neoplasm: urinary organs; multiple overlapping areas | 13 | 0,1% | 7 | 0,0% |
| C68.9 | Malignant neoplasm: urinary organ; unspecified | 37 | 0,2% | 27 | 0,2% |
| C69.3 | Malignant neoplasm: choroid | 12 | 0,1% | 5 | 0,0% |
| C71.0 | Malignant neoplasm: cerebrum; excluding cerebral lobes and Ventricle | 39 | 0,2% | 18 | 0,1% |
| C71.1 | Malignant neoplasm: Frontal lobe | 139 | 0,9% | 37 | 0,2% |
| C71.2 | Malignant neoplasm: Temporal lobe | 102 | 0,7% | 29 | 0,2% |
| C71.3 | Malignant neoplasm: Parietal lobe | 57 | 0,4% | 17 | 0,1% |
| C71.4 | Malignant neoplasm: Occipital lobe | 13 | 0,1% |  | 0,0% |
| C71.5 | Malignant neoplasm: Cerebral ventricle | 10 | 0,1% |  | 0,0% |
| C71.6 | Malignant neoplasm: Cerebellum | 5 | 0,0% |  | 0,0% |
| C71.7 | Malignant neoplasm: Brainstem | 10 | 0,1% |  | 0,0% |
| C71.8 | Malignant neoplasm: Brain; overlapping multiple subregions | 134 | 0,9% | 38 | 0,2% |
| C71.9 | Malignant neoplasm: Brain; unspecified | 116 | 0,7% | 40 | 0,3% |
| C72.8 | Malignant neoplasm: Brain and other parts of the central nervous system; overlapping multiple subregions | 7 | 0,0% |  | 0,0% |
| C73 | Malignant neoplasm of the thyroid gland | 26 | 0,2% | 15 | 0,1% |
| C76.2 | Malignant neoplasm of ill-specified sites: Abdomen | 5 | 0,0% | 5 | 0,0% |
| C76.3 | Malignant neoplasm of ill-specified sites: Pelvis | 6 | 0,0% |  | 0,0% |
| C77.0 | Secondary and unspecified malignant neoplasm: Lymph nodes of the head; face; and neck |  | 0,0% | 172 | 1,1% |
| C77.1 | Secondary and unspecified malignant neoplasm: Intrathoracic lymph nodes |  | 0,0% | 712 | 4,5% |
| C77.2 | Secondary and unspecified malignant neoplasm: Intra-abdominal Lymph nodes |  | 0,0% | 707 | 4,5% |
| C77.3 | Secondary and unspecified malignant neoplasms: Axillary lymph nodes and upper extremity lymph nodes |  | 0,0% | 168 | 1,1% |
| C77.4 | Secondary and unspecified malignant neoplasms: Inguinal lymph nodes and lower extremity lymph nodes |  | 0,0% | 102 | 0,7% |
| C77.5 | Secondary and unspecified malignant neoplasms: Intrapelvic lymph nodes |  | 0,0% | 116 | 0,7% |
| C77.8 | Secondary and unspecified malignant neoplasm: Lymph nodes of multiple regions |  | 0,0% | 334 | 2,1% |
| C77.9 | Secondary and unspecified malignant neoplasm: Lymph nodes; unspecified |  | 0,0% | 208 | 1,3% |
| C78.0 | Secondary malignant neoplasm of the lung | 25 | 0,2% | 2032 | 13,0% |
| C78.1 | Secondary malignant neoplasm of the mediastinum |  | 0,0% | 30 | 0,2% |
| C78.2 | Secondary malignant neoplasm of the pleura | 49 | 0,3% | 737 | 4,7% |
| C78.3 | Secondary malignant neoplasm of other and unspecified respiratory organs |  | 0,0% | 10 | 0,1% |
| C78.4 | Secondary malignant neoplasm of the small intestine |  | 0,0% | 33 | 0,2% |
| C78.5 | Secondary malignant neoplasm of the colon and rectum |  | 0,0% | 51 | 0,3% |
| C78.6 | Secondary malignant neoplasm of the retroperitoneum and peritoneum | 65 | 0,4% | 1680 | 10,7% |
| C78.7 | Secondary malignant neoplasm of the liver and intrahepatic bile ducts | 39 | 0,2% | 3032 | 19,4% |
| C78.8 | Secondary malignant neoplasm of other and unspecified digestive organs |  | 0,0% | 155 | 1,0% |
| C79.0 | Secondary malignant neoplasm of the kidney and renal pelvis |  | 0,0% | 70 | 0,4% |
| C79.1 | Secondary malignant neoplasm of the bladder and other and unspecified urinary organs |  | 0,0% | 25 | 0,2% |
| C79.2 | Secondary malignant neoplasm of the Skin |  | 0,0% | 218 | 1,4% |
| C79.3 | Secondary malignant neoplasm of the brain and meninges | 254 | 1,6% | 1553 | 9,9% |
| C79.4 | Secondary malignant neoplasm of other and unspecified parts of the nervous system | 6 | 0,0% | 71 | 0,5% |
| C79.5 | Secondary malignant neoplasm of the bone and bone marrow | 203 | 1,3% | 2728 | 17,4% |
| C79.6 | Secondary malignant neoplasm of the ovary |  | 0,0% | 18 | 0,1% |
| C79.7 | Secondary malignant neoplasm of the adrenal gland |  | 0,0% | 411 | 2,6% |
| C79.81 | Secondary malignant neoplasm of the mammary gland |  | 0,0% | 6 | 0,0% |
| C79.82 | Secondary malignant neoplasm of the genital organs |  | 0,0% | 14 | 0,1% |
| C79.83 | Secondary malignant neoplasm of the pericardium |  | 0,0% | 15 | 0,1% |
| C79.84 | Other secondary malignant neoplasm of the heart |  | 0,0% | 7 | 0,0% |
| C79.85 | Secondary malignant neoplasm of the connective tissue and other soft tissues of the neck |  | 0,0% | 14 | 0,1% |
| C79.86 | Secondary malignant neoplasm of the connective tissue and other soft tissues of the extremities |  | 0,0% | 26 | 0,2% |
| C79.88 | Secondary malignant neoplasm of other specified sites | 7 | 0,0% | 315 | 2,0% |
| C79.9 | Secondary malignant neoplasm of unspecified site |  | 0,0% | 46 | 0,3% |
| C80.0 | Malignant neoplasm; primary site unknown; so designated | 326 | 2,1% | 184 | 1,2% |
| C80.9 | Malignant neoplasm; unspecified | 10 | 0,1% | 12 | 0,1% |
| C81.2 | Mixed cell (classic) Hodgkin's lymphoma | 6 | 0,0% |  | 0,0% |
| C81.9 | Hodgkin's lymphoma; unspecified | 7 | 0,0% |  | 0,0% |
| C83.0 | Small B-cell lymphoma | 11 | 0,1% | 12 | 0,1% |
| C83.1 | Mantle cell lymphoma | 7 | 0,0% | 6 | 0,0% |
| C83.3 | Diffuse large B-cell lymphoma | 115 | 0,7% | 41 | 0,3% |
| C84.0 | Mycosis fungoides | 5 | 0,0% |  | 0,0% |
| C85.1 | B-cell lymphoma; unspecified | 25 | 0,2% | 14 | 0,1% |
| C85.9 | Non-Hodgkin's lymphoma; unspecified | 13 | 0,1% | 10 | 0,1% |
| C88.00 | Waldenström's macroglobulinemia: No complete remission reported |  | 0,0% | 6 | 0,0% |
| C90.00 | Multiple myeloma: No complete remission reported | 112 | 0,7% | 59 | 0,4% |
| C90.30 | Solitary plasmacytoma: No complete remission reported | 11 | 0,1% | 7 | 0,0% |
| C91.00 | Acute lymphocytic leukemia [ALL]: No complete remission reported | 6 | 0,0% | 6 | 0,0% |
| C91.10 | B-cell chronic lymphocytic leukemia [CLL]: No complete remission reported | 12 | 0,1% | 35 | 0,2% |
| C92.00 | Acute myeloblastic leukemia [AML]: No complete remission reported | 114 | 0,7% | 41 | 0,3% |
| C92.10 | BCR/ABL-positive chronic myeloid leukemia [CML]: No complete remission reported | 7 | 0,0% | 6 | 0,0% |
| C92.80 | Acute myeloid leukemia with multilineage dysplasia: No complete remission reported | 12 | 0,1% | 8 | 0,1% |
| C93.10 | Chronic myelomonocytic leukemia: No complete remission reported | 10 | 0,1% |  | 0,0% |
